# Maternal n-3 Polyunsaturated Fatty Acid Enriched Diet Commands Fatty Acid Composition in Postnatal Brain and Protects from Neonatal Arterial Focal Stroke

**DOI:** 10.1007/s12975-021-00947-9

**Published:** 2021-10-21

**Authors:** Tetyana Chumak, Matthieu J. Lecuyer, Anders K. Nilsson, Joel Faustino, Maryam Ardalan, Pernilla Svedin, Ulrika Sjöbom, Joakim Ek, Andre Obenaus, Zinaida S. Vexler, Carina Mallard

**Affiliations:** 1 Department of Physiology, Institute of Neuroscience and Physiology, Sahlgrenska Academy, University of Gothenburg, Box 432, 405 30 Gothenburg, Sweden; 2 Department of Neurology, UCSF, San Francisco, CA USA; 3 Department of Clinical Neuroscience, Institute of Neuroscience and Physiology, Sahlgrenska Academy, University of Gothenburg, Gothenburg, Sweden; 4 Institute of Health and Care Sciences, Sahlgrenska Academy, University of Gothenburg, Gothenburg, Sweden; 5 Department of Pediatrics, University of California Irvine, Irvine, CA USA

**Keywords:** PUFA, Neonatal stroke, MCAO

## Abstract

The fetus is strongly dependent on nutrients from the mother, including polyunsaturated fatty acids (PUFA). In adult animals, n-3 PUFA ameliorates stroke-mediated brain injury, but the modulatory effects of different PUFA content in maternal diet on focal arterial stroke in neonates are unknown. This study explored effects of maternal n-3 or n-6 enriched PUFA diets on neonatal stroke outcomes. Pregnant mice were assigned three isocaloric diets until offspring reached postnatal day (P) 10–13: standard, long-chain n-3 PUFA (n-3) or n-6 PUFA (n-6) enriched. Fatty acid profiles in plasma and brain of mothers and pups were determined by gas chromatography–mass spectrometry and cytokines/chemokines by multiplex protein analysis. Transient middle cerebral artery occlusion (tMCAO) was induced in P9-10 pups and cytokine and chemokine accumulation, caspase-3 and calpain-dependent spectrin cleavage and brain infarct volume were analyzed. The n-3 diet uniquely altered brain lipid profile in naïve pups. In contrast, cytokine and chemokine levels did not differ between n-3 and n-6 diet in naïve pups. tMCAO triggered accumulation of inflammatory cytokines and caspase-3-dependent and -independent cell death in ischemic-reperfused regions in pups regardless of diet, but magnitude of neuroinflammation and caspase-3 activation were attenuated in pups on n-3 diet, leading to protection against neonatal stroke. In conclusion, maternal/postnatal n-3 enriched diet markedly rearranges neonatal brain lipid composition and modulates the response to ischemia. While standard diet is sufficient to maintain low levels of inflammatory cytokines and chemokines under physiological conditions, n-3 PUFA enriched diet, but not standard diet, attenuates increases of inflammatory cytokines and chemokines in ischemic-reperfused regions and protects from neonatal stroke.

**Graphic Abstract:**

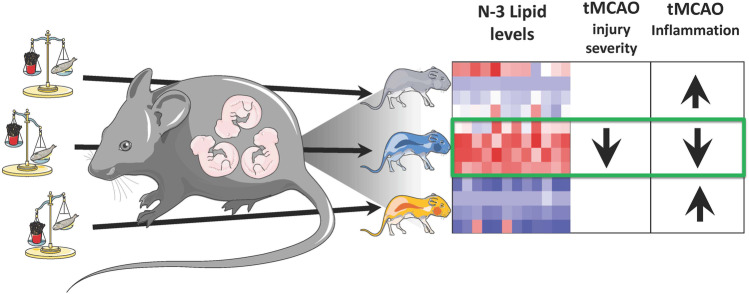

**Supplementary Information:**

The online version of this article contains supplementary material available 10.1007/s12975-021-00947-9.

## Introduction

The developing fetus is strongly dependent on nutrients from the mother, including polyunsaturated fatty acids (PUFA), and neonatal plasma PUFA levels correlate with maternal plasma and breast milk concentrations [1, 2]. The brain is highly enriched in n-6 PUFA arachidonic acid (AA, 20:4 n-6) and n-3 PUFA docosahexaenoic acid (DHA, 22:6 n-3) [3], and the balance of n-3:n-6 PUFA is critical for brain development [4]. Both n-3 and n-6 long-chain (LC) PUFA are essential components of cell membranes but are also precursors to bioactive lipid mediators. In extremely preterm infants, lower serum levels of DHA at birth are associated with inflammatory responses postnatally [5]. DHA is a precursor of several metabolites that regulate resolution of inflammation [6], while n-6 metabolites are considered mostly proinflammatory [7]. Nutritional supplementation during pregnancy demonstrates that maternal diets enriched in n-3 PUFA reduce inflammatory responses in the placenta [8] and prevent LPS-induced cytokine production in offspring microglial cells [9].

Brain injury in the newborn is diverse and constitutes a significant clinical problem [10]. Perinatal arterial ischemic stroke is estimated to occur in 1 in 3,500 full-term infants [11] resulting in numerous long-term neurological sequelae [12]. Neuroinflammation is an important factor in the pathogenesis of neonatal stroke [13, 14], however many aspects of the underlying mechanisms remain unclear and interventions to improve outcome are limited and no pharmacotherapies exist. While in adult rodents n-3 PUFA ameliorates neuroinflammation and mitigates ischemic stroke damage through interactions with astrocytes and microglia [15], the effect of n-3/n-6 maternal diets on offspring inflammatory signaling and neuronal protection following neonatal stroke is unknown. However, a study that utilized MRI to examine white matter injury in preterm human infants showed association between higher DHA levels and reduced intraventricular hemorrhage damage, but other fatty acids did not produce such an effect [16]. Strikingly, just 1% higher PUFA levels in the blood were associated with 4.3-fold decreased odds ratio of injury and higher language and motor scores, suggesting a critically important role of n-3 PUFA levels during gestation for resilience of the immature brain to injury [16].

Thus, in the present study we investigated the role of LC n-3 PUFA enriched diet during gestation and early postnatal life on the naïve brain and after neonatal stroke. We show that maternal n-3 PUFA enriched diet under physiological conditions dictates unique brain lipid composition without major effects on cytokine and chemokine levels, but it attenuates neuroinflammation induced by transient middle cerebral artery occlusion (tMCAO) and provides a neuroprotective milieu for neonatal stroke.

## Materials and Methods

### Animals

C57BL/6J mice were given ad libitum access to food and water, housed with nesting material and shelters, and kept in rooms with temperature control and light/dark cycles. All experiments were performed in accordance with ethical protocols approved by the Gothenburg Animal Ethics Committee or UCSF’s Institutional Animal Care and Use Committee and in accordance to the Guide for the Care and Use of Laboratory Animals (U.S. Department of Health and Human Services). The data are in compliance with the ARRIVE guidelines (Animal Research: Reporting in Vivo Experiments).

### Diets and Breeding

In order to investigate the impact of the balance of n3:n6 PUFA on brain physiology and outcome following stroke during the neonatal period, three diets were studied. Breeding cages were randomly assigned to one of the experimental isocaloric diets (AIN-93G, BioServ): standard (Stand, BioServ #S7628;), n-3 LC-PUFA enriched (n-3, BioServ #S7630; soybean oil replaced with Menhaden Fish oil) or n-6 enriched (n-6, BioServ #S7629; based on corn oil). The n-3:n-6 ratio in diets was: standard 0.13; n-3 enriched 1.32; n-6-enriched 0.02. The AA/DHA ratio for n-3 enriched diet was 0.14, while AA and DHA levels in standard and n-6 enriched diets were below 0.01% and the ratio not calculated. Diet composition is listed in Table 1. The day of birth was defined as postnatal day (P) 0. After giving birth, the dams remained on the experimental diet until offspring sacrifice (P10-P12). To decrease litter effects, study groups were distributed across 7–8 litters for each diet (n = 10–12 pups per diet). Similar number of pups of both sexes were included in each experimental group.

**Table 1:** Fatty acid profile in food

Fatty Acid Class	Stand.,%	n-3, %	n-6, %
Alpha Linolenic (18:3 n3)	0.318	0.113	0.049
Dihomolinolenic (20:4 n3)	<0.01	0.038	<0.01
Eicosapentaenoic (20:5 n3)	<0.01	0.412	0.013
Docosapentenoic (22:5 n3)	<0.01	0.068	<0.01
Docosahexanoic (22:6 n3)	<0.01	0.259	<0.01
Linoleic (18:2 n6)	2.37	0.707	2.56
Arachidonic (20:4 n6)	<0.01	0.036	<0.01
Butyric (4:0)	<0.01	<0.01	<0.01
Caproic (6:0)	<0.01	<0.01	<0.01
Caprylic (8:0)	<0.01	<0.01	<0.01
Capric (10:0)	<0.01	<0.01	<0.01
Lauric (12:0)	<0.01	<0.01	<0.01
Myristic (14:0)	0.013	0.276	0.018
Pentadecanoic (15:0)	<0.01	0.021	<0.01
Palmitic (16:0)	0.551	0.683	0.621
Heptadecanoic (17:0)	<0.01	0.016	<0.01
Stearic (18:0)	0.188	0.142	0.094
Arachidic (20:0)	0.015	<0.01	0.017
Behenic (22:0)	0.015	<0.01	<0.01
Myristoleic (14:1)	<0.01	<0.01	<0.01
Palmitoleic (16:1)	<0.01	0.411	0.017
Oleic (18:1)	1.08	0.454	1.35
Saturated Fatty Acids	0.747	1.08	0.715
Monounsaturated Fatty Acids	1.12	0.97	1.36
Polyunsaturated Fatty Acids	2.57	1.67	2.51
n-3 Fatty Acids	0.318	0.992	0.06
n- 6 Fatty Acids	2.37	0.754	2.56

### Fatty Acid Analysis

Total lipid extraction was based on the BUME method for plasma [17] and animal tissue [18]. The plasma phospholipids or brain total lipids were subjected to acid transesterification for the preparation of fatty acid methyl esters and analyzed on an Agilent 7820/5975 GC–MS system (Agilent Technologies, Palo Alto, CA, US). Fatty acid methyl esters were identified based on the retention times and mass spectra of authentic standards (Larodan, Solna, Sweden). Quantification was performed in Agilent MassHunter Workstation Quantitative analysis software Version 10.0 (Agilent Technologies). Fatty acids are expressed as weight% of all analyzed fatty acids.

### Cytokine Multiplex Assays

Cytokine/chemokine levels were measured in the blood plasma and brain tissue of saline perfused P10 naïve mice using Bio-Plex Pro Mouse Cytokine Standard 31-Plex kit (Cat# 12,009,159, BioRad) on a Bio-Plex 200 analyzer according to the manufacturer's instructions. Cytokine/chemokine levels of mice that underwent tMCAO were measured using 23-plex cytokine kit (Cat# M60009RDPD, Biorad), principally as we previously reported [19–21].

### Western Blot

Whole cortical lysates from ischemic-reperfused and matching contralateral regions were homogenized and proteins separated on SDS–polyacrylamide gels (Invitrogen Life Technologies). The membranes were incubated with an anti-spectrin and anti-actin antibodies (1:2000, EMD Millipore Corporation).

### Transient MCAO in Neonatal Mice and Injury Volume

Transient 3-h MCAO was performed using the Derugin model in P9-P10 pups, as previously described [19, 22]. Briefly, a nylon suture was advanced 4–5 mm into the common carotid artery and removed 3 h later. Brains were collected at 72 h from saline- perfused and paraformaldehyde fixed mice. Cryoprotected brains were coronally sectioned at 12 µm (every 350 µm). Volume of injury was measured in Nissl-stained brains by ImageJ software on eight slices per brain.

### Data and Statistical Analysis

Statistical analysis was performed using IBM SPSS Statistics 25 (IBM Corp, Armonk, NY, United States) and Graphpad PRISM 8 (GraphPad Software, San Diego, CA). Data with normal distribution was analyzed by one-way ANOVA with Tukey post-hoc test or Welch’s ANOVA with Dunnett’s post hoc test when groups had unequal variances, and non-normal distribution data were analyzed by Kruskal–Wallis test followed by Dunn’s post hoc test. Two-way ANOVA was used to test the interacton between independent variables (diet and sex, diet and injury) and main effects of diet and sex on fatty acid and cytokine levels and diet and injury on spectrin and cytokine levels. Differences were considered statistically significant at *p* < 0.05. Fatty acid data are presented as median with quartiles and at 10–90th percentile. Cytokine and brain injury data are presented as median with quartiles and whiskers or mean ± SD as indictaed in figure legends. Graphs also include plotting of individual data points. Prinicipal component analysis (PCA) and heatmaps were generated using Qlucore Software (Lund, Sweden).

## Results

### Maternal Diet Dictates Fatty Acid Composition in Neonatal Brain

All breeders, independent of diet, showed similar weight gain over the course of feeding (Suppl. Figure 1a) and number of pups per litter was not affected by diet (Suppl Fig. 1b). However, pups born to the mothers fed n-3 diet were slightly heavier in absolute weight at P10 compared to pups born to mothers fed the other two diets (Suppl. Figure 1c).

**Fig. 1:**
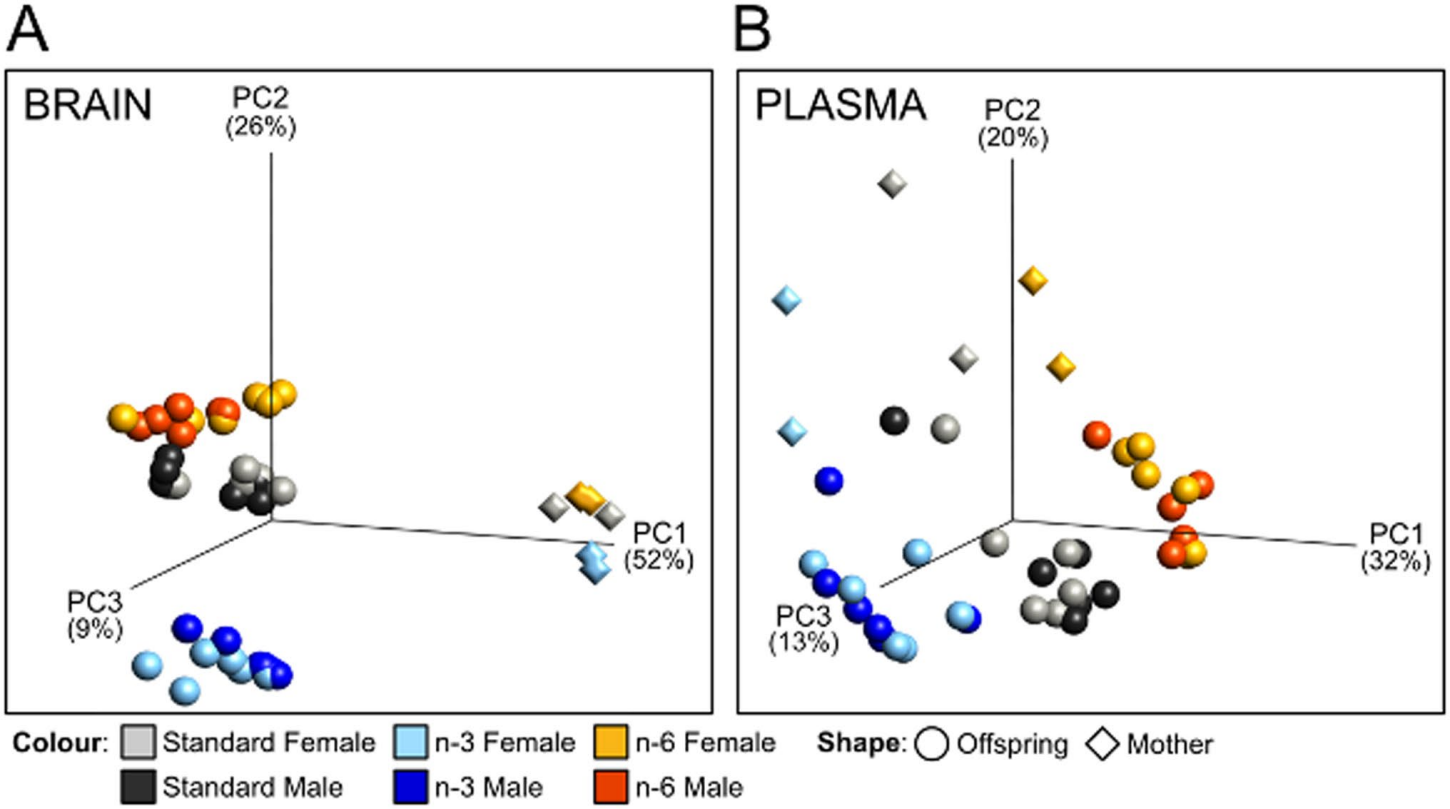
Principal component analysis of fatty acid composition in brain and blood plasma. 3D Principal component analysis plots showing distribution of samples based on composition of fatty acids in brain (**a**) and blood plasma (**b**) of pups and dams fed three different diets. The plots show clustering of samples in all dimensions (diet, tissue and generation). Colour depictions for standard diet: grey for females, black for males; n-6 diet: yellow for females, red for males; n-3 diet: light blue for females, dark blue for males. Mothers (n = 2/diet) are depicted by diamonds and pups (n = 11–12/diet) by circles

The total amount of fatty acids per gram brain tissue was similar in P10 pups independent of diet (Stand.: 41.32 ± 4.32; n-3: 47.43 ± 4.83, n-6: 43.76 ± 6.60; *p* > 0.05). Non-hierarchic grouping using dimension reducing principal component analysis (PCA), in turn, showed that maternal diet markedly affected clustering of fatty acids in a n-3–specific manner in neonatal brain tissue compared to pups from mothers on standard and n-6 diets (Fig. 1a). In the mothers, brain fatty acid composition was markedly less dependent on diet and distinctly different from pups (Fig. 1a). In plasma, PCA of fatty acids discriminated mothers and pups into respective diet groups, but to a lesser extent than in brain (Fig. 1b).

### Maternal Diet Alters Polyunsaturated Fatty Acid Content in Neonatal Plasma

**Fig. 2:**
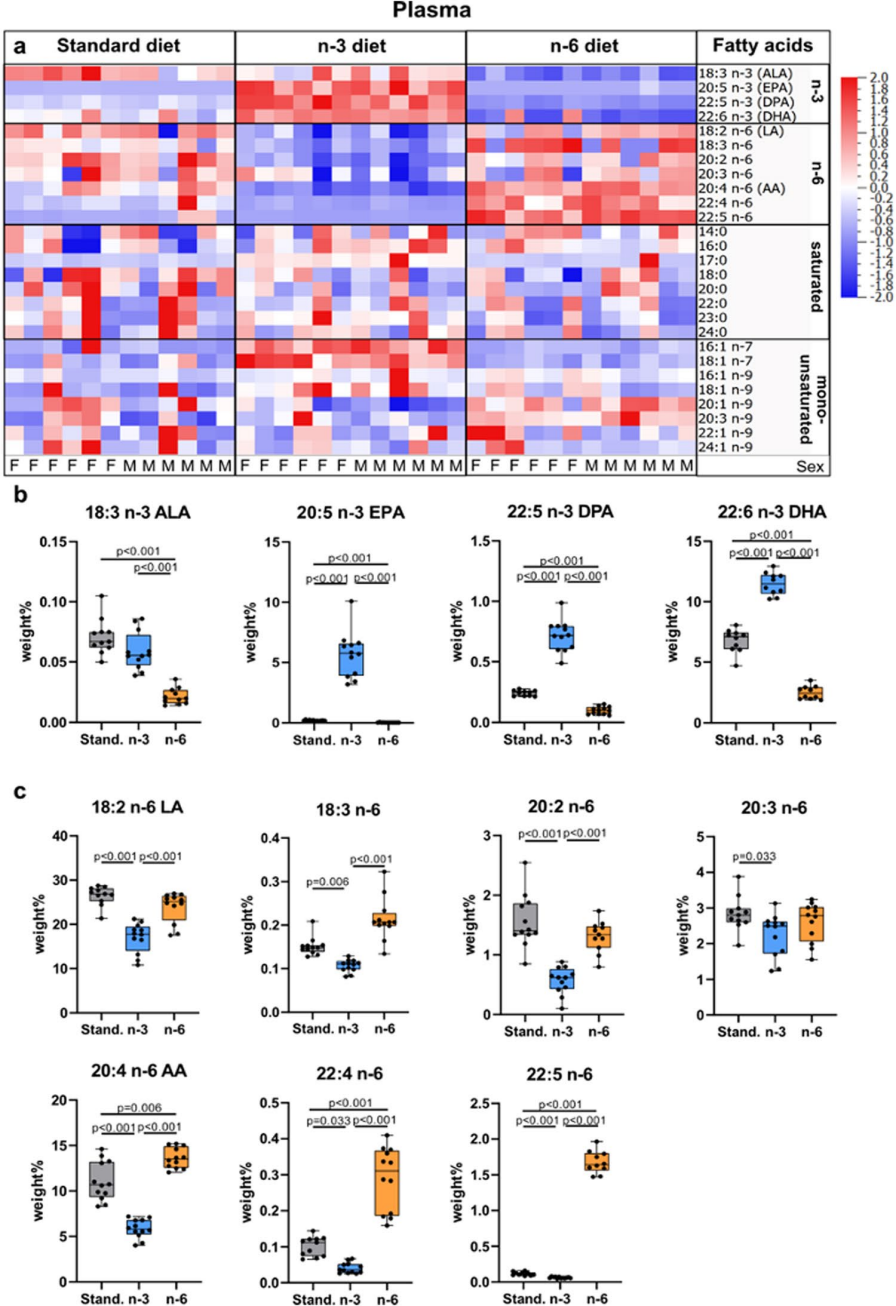
Phospholipid fatty acid composition in blood plasma of offspring. Heat map of fatty acids analyzed in blood plasma of offspring from mothers fed a standard, n-3 enriched or a n-6 enriched diet. Each column refers to one animal and each row shows levels of a specific fatty acid. Each fatty acid level was normalized over all samples using *Z*-score normalization and is shown using a colour scheme based on *Z*-score distribution from -2 to 2. Sex of pups as indicated: F for females and M for males. (**a**) Levels of n-3 fatty acids in the blood plasma of offspring from mothers fed a standard (Stand.), n-3 enriched or a n-6 enriched diet (**b**). Levels of n-6 fatty acids in blood plasma of offspring from mothers fed a standard, n-3 enriched or a n-6 enriched diet (**c**). Data are presented as median with quartiles and whiskers representing range as well as plotting individual data (n = 11–12/diet). One-way ANOVA with post-hoc Tukey’s test, Welch’s ANOVA with post hoc Dunnett’s test or Kruskal–Wallis with post-hoc Dunn’s multiple comparison test was performed to compare groups. Individual *p*-values are listed within figures for data that are significantly different

Measurements of fatty acid levels in the plasma showed that the n-3 diet results in enrichment of n-3 fatty acids in pups whereas n-6 fatty acid increase occurs in pups from mothers fed the n-6 diet (Fig. 2a, Suppl. Table 1). At the same time, we found lower levels of all n-3 fatty acids (ALA, EPA, n-3 Docosapentaenoic acid [n-3 DPA] and DHA) in blood plasma of pups from mothers fed n-6 diet compared to standard and n-3 diets (Fig. 2b). All plasma n-6 fatty acids measured were lower in pups from mothers fed n-3 diet compared to standard diet (Fig. 2c). The n-3:n-6 ratio in plasma was 0.21 ± 0.09 in standard diet, 0.77 ± 0.25 in n-3 diet (*p* < 0.001 vs standard) and 0.08 ± 0.04 in n-6 diet (*p* = 0.17 vs standard; *p* < 0.001 vs n-3). The DHA level in neonatal blood plasma was 78% lower in pups from n-6 diet and 41% lower in standard diet compared to n-3 fed pups. There were no changes in saturated fatty acids except 17:0 which increased in plasma in n-3 pups compared to the two other diets (Fig. 2a, Suppl. Table 1), while monounsaturated fatty acids (MUFAs) 18:1 n-7, 18:1 n-9, and 20:1 n-9 were increased in n-3 diet compared to standard diet and 18:1 n-9 was increased with n-6 diet (Suppl. Table 1). We found no effect of sex on plasma levels of fatty acids except 20:3 n-9 showing increased level in plasma of male pups fed n-6 diet compared to standard diet (p = 0.03), but not in females (Suppl. Figure 2a).

### Maternal Diet Alters Polyunsaturated Fatty Acid Content in Neonatal Brain

**Fig. 3:**
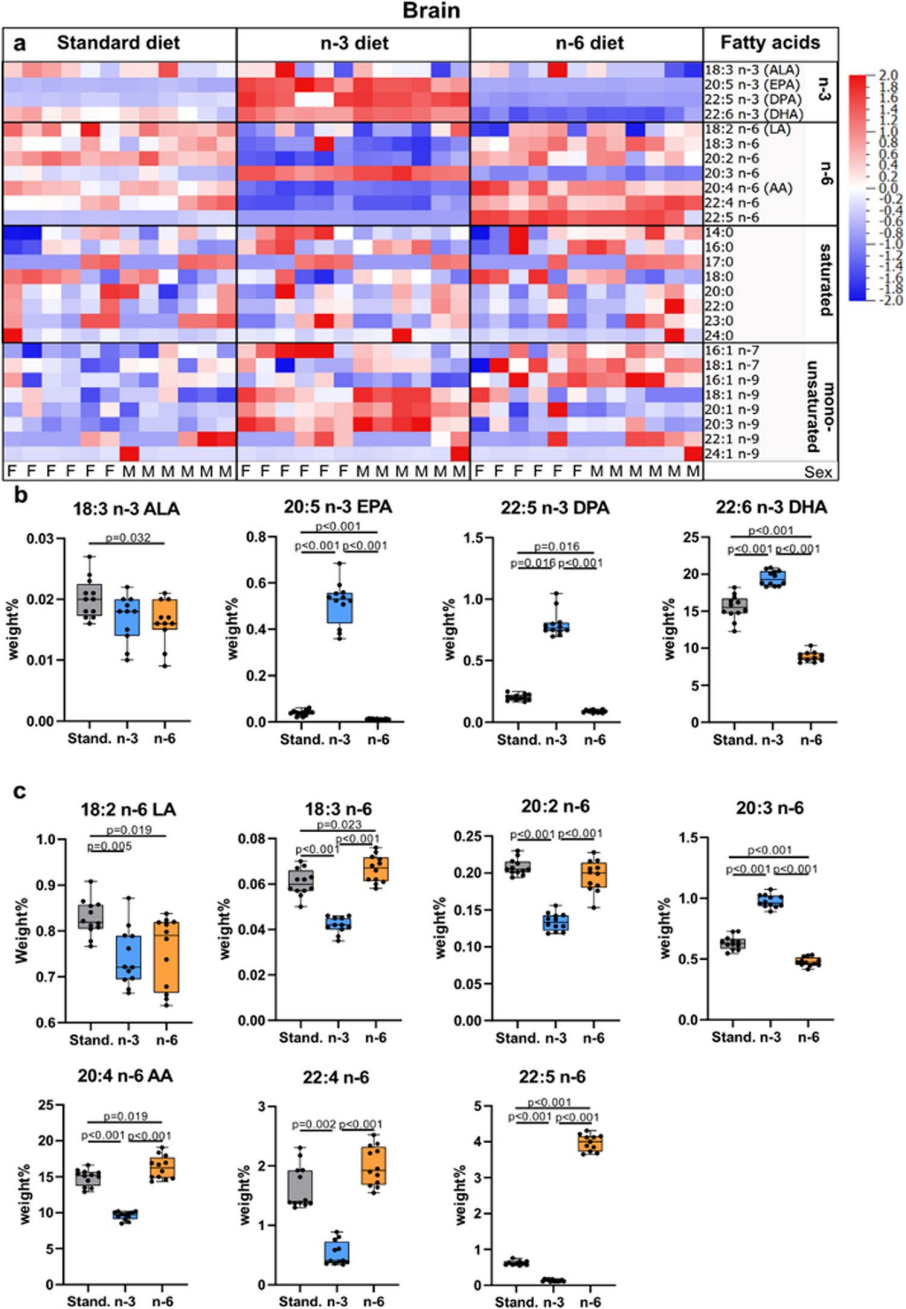
Total lipid fatty acid composition in brain of offspring. Heat maps of fatty acids analyzed in brain of offspring from mothers fed a standard (Stand.), n-3 enriched or a n-6 enriched diet. Each column refers to one animal and each row shows levels of a specific fatty acid. Each fatty acid level was normalized over all samples using *Z*-score normalization and is shown using a colour scheme based on Z score distribution from -2 to 2. Sex of pups as indicated: F for females and M for males. (**a**) Levels of n-3 fatty acids in the brain of offspring from mothers fed a standard, n-3 enriched or a n-6 enriched diet (**b**). Levels of n-6 fatty acids in brain of offspring from mothers fed a standard, n-3 enriched or a n-6 enriched diet (**c**). The data are presented as median with quartiles and whiskers representing range as well as plotting individual data (n = 11–12/diet). One-way ANOVA with post-hoc Tukey’s test, Welch’s ANOVA with post hoc Dunnett’s test or Kruskal–Wallis with post hoc Dunn’s multiple comparison test was performed to compare groups. Individual *p*-values are listed within figures for data that are significantly different

As evident from heatmaps, n-3 diet resulted in enrichment of n-3 fatty acids and decrease in n-6 fatty acids in the offspring brain compared to pups from mothers fed the standard diet (Fig. 3a, Suppl. Table 2). Conversely, n-6 fatty acids were increased and n-3 fatty acids decreased in brains of pups on the n-6 diet (Fig. 3a, Suppl. Table 2). Specifically, levels of EPA, n-3 DPA, and DHA were higher in pups whose mothers were fed n-3 diet compared to standard diet (Fig. 3b). Similarly to plasma, brain of pups from mothers fed standard and n-6 diet contained lower DHA than in the brain of pups fed n-3 diet, 80% and 46% of the levels, respectively. All measured n-6 fatty acids were decreased in pups whose mothers were fed n-3 diet, with the exception of 20:3 n-6 (Dihomo-γ-linolenic acid, DGLA), which was increased (Fig. 3c). n-3 PUFA as well as n-6 PUFA linoleic acid (LA, 18:2 n-6) and DGLA levels were decreased in the brain of pups from mothers fed n-6 diet compared to standard diet (Fig. 3b, c). The n-3:n-6 ratio in brain was 0.83 ± 0.09 in standard, 1.76 ± 0.11 in n-3 diet (*p* < 0.001 vs standard) and 0.39 ± 0.05 in n-6 diet (*p* < 0.001 vs standard; *p* < 0.001 vs n-3).

Among the saturated fatty acids, the n-3 diet decreased 24:0 while no changes were apparent with n-6 diet compared to standard diet. Among the MUFAs, the n-3 diet increased 16:1 n-7, 18:1 n-9, 20:1 n-9, and 20:3 n-9, while n-6 diet increased levels of 16:1 n-9, 16:1 n-7, and 18:1 n-7 compared to standard diet (Fig. 3a, Suppl. Table 2). Levels of fatty acids in the brain were not affected by sex.

### Maternal Diet Affects Cytokine/Chemokine Profile in the Neonatal Brain but not in the Blood

**Fig. 4:**
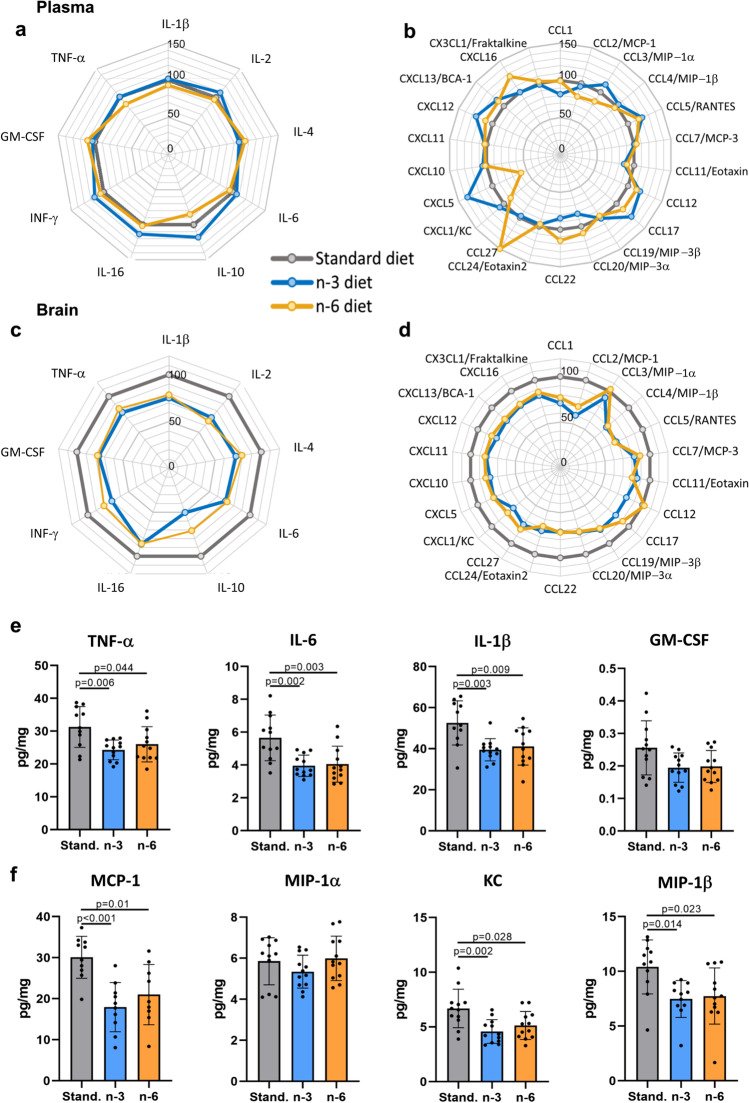
Cytokines and chemokines in blood and brain of offspring. Radar charts showing blood plasma levels of cytokines (**a**) and chemokines (**b**) and brain levels of cytokines (**c**) and chemokines (**d**) in pups born to mothers fed a n-3 (blue line) or n-6 (yellow line) enriched diet in relation to levels in the tissues of pups whose mothers were fed standard diet (grey line, level set to 100%). Levels of specific cytokines (**e**) and chemokines (**f**) in the brain of offspring from mothers fed a standard (grey), n-3 enriched (blue) or a n-6 enriched (yellow) diet. Data presented as mean ± SD. One-way ANOVA with post-hoc Tukey’s multiple comparison test was performed to compare groups. Individual p-values are listed within figures for data that are significantly different. N = 10–12/diet

None of the 31 cytokines and chemokines measured in plasma from naïve pups were altered in n-3 and n-6 compared to standard diet (Fig. 4a, b; Suppl. Table 3). In contrast, in brain of pups from n-3 diet compared to standard diet pups concentrations of 7/9 cytokines and 16/22 chemokines were reduced (Fig. 4c, d, Suppl. Table 4). In pups from n-6 diet, brain levels of 6/9 cytokines and 12/22 chemokines were lower compared to pups from standard diet. Cytokine and chemokine levels in plasma were not different between male and female pups, while levels of Eotaxin 2 and CCL22 were affected by diets to higher extent in brains of females compared to males (Suppl. Figure 2b).

The levels of inflammatory mediators often implicated in neonatal brain injury, including pro-inflammatory cytokines TNF-α, IL-6 and IL-1β (Fig. 4e) and chemokines MCP-1, KC and MIP-1β (Fig. 4f), were lower in brains in pups from both n-3 and n-6 diets compared to standard diet.

### Maternal n-3 Enriched Diet Protects Neonatal Mice from tMCAO

**Fig. 5:**
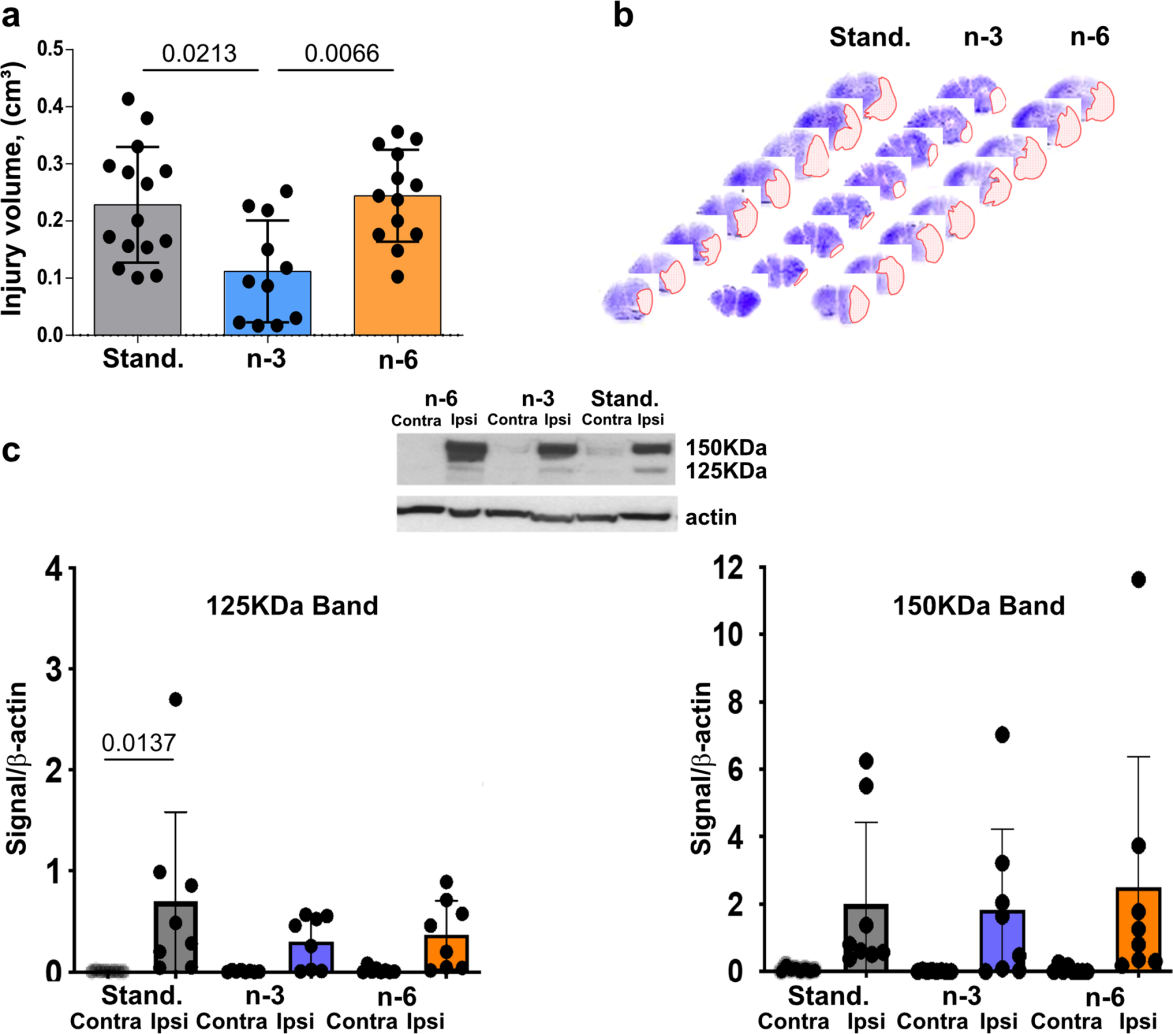
Effects of maternal diet on neuroprotective responses following tMCAO. (**a**) Volume of brain injury 72 h after tMCAO reperfusion in pups born to mothers fed a standard (grey), n-3 (blue) or n-6 (yellow) enriched diet. (**b**) Representative examples of anterior-to-posterior injury distribution in neonatal brain following tMCAO (Nissl staining). (**c**) Spectrin cleavage 24 h after reperfusion. Western Blot shows representative examples of spectrin cleavage via caspase-3-dependent (125KDa band, left) and calpain-dependent (150KDa band, right) in injured (ipsi) and non-injured (contra) brain cortex. Data presented as mean ± SD. Kruskal–Wallis test followed by Dunn’s post-hoc test (**a**) or two-way ANOVA followed by Tukey’s post hoc test (**c**) was performed to compare groups. Individual p-values are listed within figures for data that are significantly different. N = 7–15/diet

In neonatal mice subjected to tMCAO, neuropathological analysis 72 h after reperfusion demonstrated the presence of injury in all groups (Fig. 5a–b) but volume of injury was significantly lower in pups from mothers fed the n-3 diet compared to injury volumes in pups from mothers fed standard or n-6 diets (Fig. 5a). Brain injury volume was predominantly reduced in male pups (Suppl. Figure 3). To further examine injury after tMCAO we analyzed activation of caspase-3 and calpain by determining spectrin cleavage by these proteases. In each group there was significantly increased spectrin cleavage by each of proteinase in the ipsilateral side compared to respective contralateral regions (not shown). Multifactorial analysis showed that in pups from the standard diet group, caspase-3-dependent spectrin cleavage was higher in the ischemic-reperfused region compared to that in respective contralateral regions 24 h after tMCAO (125 kDa band, Fig. 5c). Although caspase-3-mediated spectrin cleavage in pups on n-3 and n-6 diets was apparent in injured regions, there was no significant difference compared to that in respective contralateral regions by multifactorial analysis (Fig. 5c). Increased calpain-dependent spectrin cleavage at 24 h was evident in injured compared to contralateral region in each of the three groups (150 kDa band, Fig. 5c). Thus, n-3 enriched diet was required to preserve neurons as feeding standard diet during gestation and postnatal period was not sufficient to protect pups from stroke.

### Maternal n-3 Enriched Diet Significantly Reduces Accumulation of Cytokines and Chemokines in Injured Brain Regions 24 h After tMCAO

**Fig. 6:**
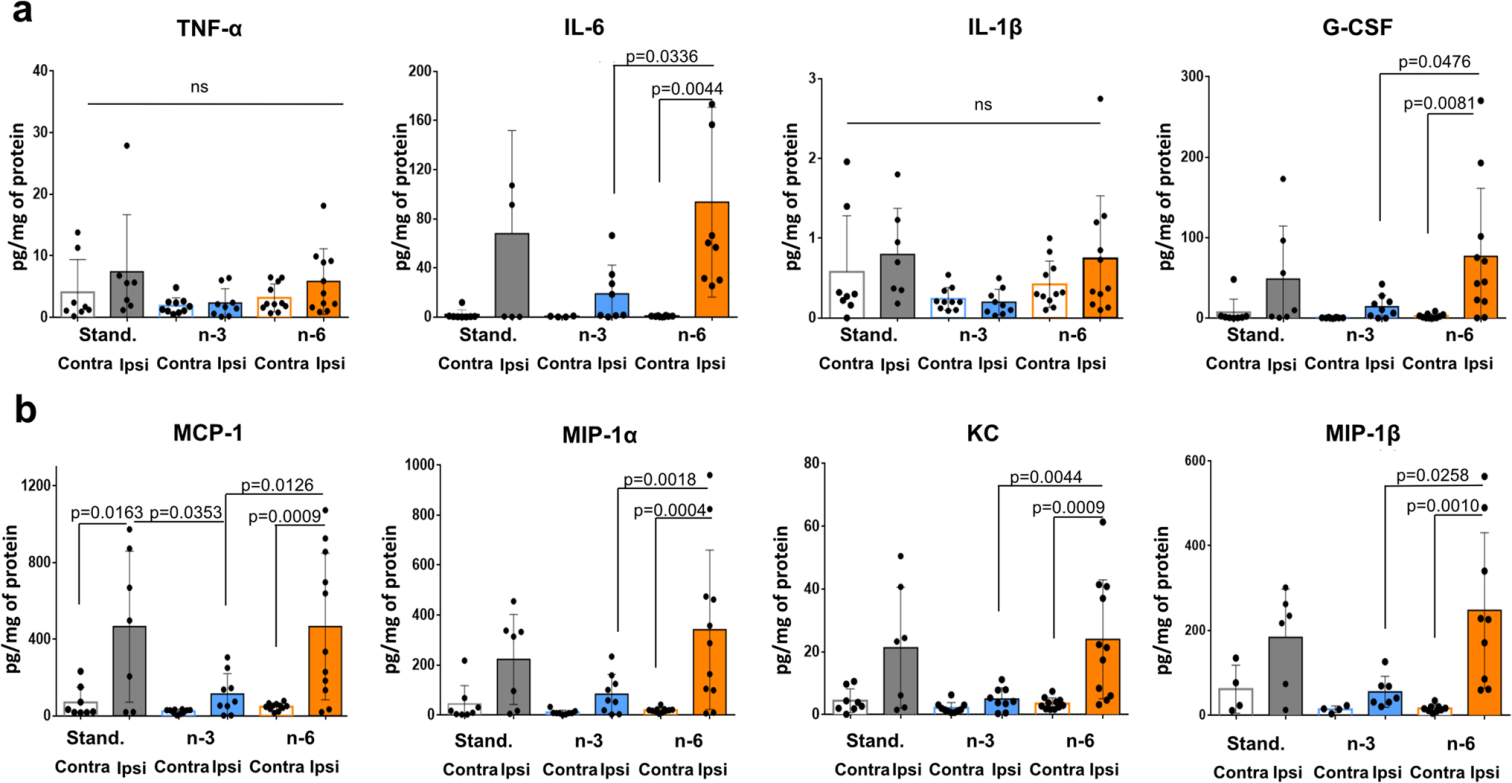
Levels of cytokines and chemokines in ischemic-reperfused cortex 24 h after neonatal tMCAO in neonatal mice. Levels of cytokines (**a**) and chemokines (**b)** in injured (ipsi) and non-injured (contra) cortex in pups born to mothers fed a standard (grey), n-3 (blue) or n-6 (yellow) enriched diet. Data are shown as mean ± SD. Statistical significance levels are as indicated on individual panels. Two-way ANOVA with post-hoc Tukey's multiple comparisons test was performed to compare groups. Individual p-values are listed within figures for data that are significantly different. N = 7–11/diet

At 24 h after tMCAO, TNF-α and IL-1β levels were not significantly increased in ischemic-reperfused brain regions compared to levels in contralateral region of pups on standard diet, consistent with our previous observations [19, 20]. Furthermore, the levels of these cytokines were not altered in injured regions in pups on any diets (Fig. 6a). In contrast, pups from mothers fed the n-6 diet demonstrated significantly increased levels of several cytokines in injured regions, including IL-6, G-CSF (Fig. 6a), and chemokines MCP-1, MIP-1α, KC and MIP-1β (Fig. 6b). Maternal n-3 enriched diet prevented accumulation of IL-6, G-CSF, MIP-1α, KC and MIP-1β in the injured region compared to n-6 diet (Fig. 6a, b). These data demonstrate that n-6 diet is not sufficient to maintain low levels of inflammatory cytokines and chemokines following stroke. N-3 diet, in turn, attenuates increases of inflammatory cytokines and chemokines in ischemic-reperfused regions.

## Discussion

Here we show that maternal n-3 enriched diet protects pups from neonatal stroke compared to pups from mothers on standard or n-6 enriched diets. We demonstrate that maternal n-3 enriched diet markedly and uniquely rearranges lipid composition in naïve neonatal brains, resulting in increased levels of n-3 LC-PUFAs and suppressed levels of most n-6 fatty acids. At the same time, n-3 supplementation was not accompanied by any specific changes in brain levels of inflammatory mediators compared to n-6 diet. In contrast, accumulation of inflammatory cytokines and chemokines induced by tMCAO was significantly attenuated in pups on n-3 diet, revealing that while the effects of n-3 diet on inflammation are not apparent under basal conditions, n-3 mediated anti-inflammatory and homeostatic effects reduce susceptibility to neonatal stroke.

During pregnancy, the placenta transports PUFA from the mother to the fetus [23] as the enzymes that metabolize PUFA are low before birth, thus, the fetus is fundamentally dependent on placental transfer of n-3 and n-6 fatty acids [24]. Neonatal plasma PUFA levels correlate with maternal plasma and breast milk concentrations in humans [2, 25]. Consistently, the lipid compositions of our maternal diet regimens are mostly reflected in neonatal plasma and produce distinct and robust changes in lipid brain content.

Most PUFAs in CNS accumulate during development [26] and, as previously reported in young adult rats, we find that DHA and AA are the most abundant PUFA in neonatal brains [27]. While the main source of DHA and AA is from food intake, the capacity of body tissues to synthesize n-3 (e.g. DHA) and n-6 (e.g., AA) LC-PUFAs from respective shorter precursors α-linolenic acid (ALA, 18:3 n-3) and linoleic acid (LA, 18:2 n-6) in amounts sufficient to supply the brain remain uncertain [28] [29]. In the current study, there were low levels of DHA and AA in standard and n-6 maternal diets, but these fatty acids were still relatively high in neonatal plasma and brain suggesting synthesis from shorter-chain precursors supplied from the mother [29]. In line with the notion that the composition of the food commands the PUFA content, there were markedly increased EPA, n-3 DPA and DHA levels, concomitant with decreased levels of most n-6 PUFA, in pups from mothers on the n-3 enriched diet.

PCA plots demonstrate well-separated diet-dependent clustering of fatty acids, particularly in neonatal brains, which is likely the result of greater extent of incorporation of fatty acids in cell membranes in the growing brain compared to the adult [30]. Such strong discrimination of brain lipids in n-3 pups, compared to pups from n-6 and standard diets, suggests that the n-3 enriched maternal supplement stipulates a unique lipid brain profile in the neonates, which concur with findings of PUFA diet modulating microglia lipid content in the offspring [31]. While most lipids in the brain followed a similar pattern to that seen in plasma, interestingly the level of n-6 DGLA was completely reversed in plasma and brain with increased levels in brain in pups from the n-3 diet. The latter finding is consistent with observations of higher DGLA in brain of mice on a standard n-3 maternal diet [32], and lower levels in mice on high-fat diet [33].

In adult rodents, neuroprotective and neurorestorative effects of n-3 PUFA have been firmly established in experimental stroke [34–38] and traumatic brain injury [39], effects that resulted in enhanced angiogenesis [40], neurogenesis [41] and preservation of white matter integrity [42]. Recently, short-chain fatty acids, derived from the gut microbiome, were shown to improve post-stroke recovery in adults via immunological mechanisms, in part mediated via microglia cells [43]. Enriched maternal n-3 diet also reduced brain injury following neonatal hypoxia–ischemia [44]. Beneficial effects in neonatal animals were attributed to anti-inflammatory and anti-oxidative properties [45] and reduced permeability of the blood–brain barrier [46]. We provide evidence that n-3 enriched diet limits cell death and induces neuroprotection in neonatal stroke accompanied by decreased caspase-3 dependent cleavage of spectrin and alleviated inflammatory response following neonatal tMCAO with both reduced expression of cytokines (IL-6 and G-CSF) and chemokines (MCP-1, MIP-1α, KC, MIP-1β). IL-6 is a known early modulator in adult stroke [47]. G-CSF and chemokines are well-known immune cell chemo-attractants and blocking monocyte-microglia signaling and diminishing neutrophil adhesion/infiltration reduces injury in an animal model of childhood stroke [48]. Additionally, caspase-3 is one of the major cell death pathways in neonatal brain injury [49]. Thus, the n-3 diet provides beneficial effects by reducing both cell death and inflammatory mechanisms following neonatal stroke.

Neuroinflammation could be a consequence of failure to cap inflammation and restore tissue homeostasis by specialized pro-resolving lipid mediators that are derived metabolically from n-3 and n-6 fatty acids. Functional significance of selective DHA enrichment in cellular membrane phospholipids was shown by upregulation of several classes of DHA-derived lipid mediators such as Neuroprotectin D1 (NPD1), Resolvin D1 (RvD1), protectins and marisins. NPD1 is anti-apoptotic and acts as a pleiotropic modulator of inflammation and injury resolution [50] and NPD1 administration reduces stroke volume after MCAO [34]. RvD1, a member of a family of potent lipid mediators derived from both EPA and DHA [51], reduces transendothelial migration of neutrophils and promotes the resolution of the inflammatory response. As such, NPD1 and RvD1 can counter-regulate proinflammatory mediators and promote uptake of cellular debris when administered short-term and in pharmacological doses.

Consistent with the notion that the ratio of n-3:n-6 is a critical factor in neuronal outcomes, we observed an increased n3:n6 ratio in the neuroprotective n-3 diet compared to standard and n-6 diets. A study using transgenic mice with increased n-3:n-6 fatty acid ratio showed protection against cognitive deficits induced by an immune challenge via decreased neuroinflammation [52]. Another line of evidence of a homeostatic role of n-3:n-6 ratio comes from studies in the *mfat‐1* transgene mouse model demonstrating that increased n-3:n-6 ratio led to protection of neural stem cells against hypoxia [53].

Nutritional n-3 PUFA deficiency during the perinatal period alters the brain innate immune system and neuronal plasticity-associated genes in hippocampus [54]. Effect of n-3 PUFA deficiency on levels of cytokine gene expression was not evident at birth but manifested at weaning age, while microglia exhibited signs of activation already at birth. A recent study also showed that maternal dietary n-3 PUFA deficiency increases microglia-mediated phagocytosis of synaptic elements in the rodent developing hippocampus, partly through the activation of 12/15-lipoxygenase (LOX)/12-HETE signaling, altering neuronal morphology and affecting cognitive performance of the offspring [55]. These findings provide mechanistic insights into neurodevelopmental defects caused by maternal n-3 PUFA dietary deficiency. A translationally important question is whether the damaging role of n-3 PUFA deficiency/insufficiency in the fetus can be overcome by boosting n-3 PUFA in the diet later in postnatal development. While literature is sparse, some studies suggest the existence of a “window” of opportunity for correction. For example, a chronic ALA deficient diet altered lipid composition of cerebral membranes and adversely affected dopaminergic neurotransmission in the developing rat brain. Reversal of n-3 PUFA-deficient diet, with adequate levels of n-6 and n-3 PUFAs during the lactating period restored both the fatty acid composition of brain membranes and dopaminergic neurotransmission by young adulthood whereas diet enrichment after weaning led to only partial recovery of biochemical parameters but no recovery of neurochemical factors [56].

In summary, we show that maternal n-3 enriched diet provides a unique brain lipid profile in the developing offspring brain, and while a standard n-3:n-6 diet is insufficient to reduce susceptibility of the neonatal brain to stroke, the n-3 enriched diet is needed to enhance brain homeostasis and improve neuroimmune status in injured neonatal brain.

## Supplementary Information

Below is the link to the electronic supplementary material.

Electronic supplementary material 1 (DOCX 15 kb)

Electronic supplementary material 2 (DOCX 43 kb)

Electronic supplementary material 3 (TIFF 368 kb)

Electronic supplementary material 4 (TIFF 2197 kb)

Electronic supplementary material 5 (TIFF 2197 kb)

## Data Availability

The data that support the findings of this study are available from the corresponding author, [CM], upon reasonable request.

## Abbreviations

AA: Arachidonic acid
ALA: α-Linolenic acid
DHA: Docosahexaenoic acid
DGLA: Dihomo-γ-linolenic acid
DPA: Docosapentaenoic acid
IVH: Intraventricular hemorrhage
LA: Linoleic acid
LC: Long-chain
MUFA: Monosaturated fatty acids
NPD1: Neuroprotectin D1
P: Postnatal day
PCA: Prinicipal component analysis
PUFA: Polyunsaturated fatty acids
RvD1: Resolvin D1
tMCAO: Transient middle cerebral artery occlusion

## Code Availability

N/A.
