# Supplementary material for: Maternal n-3 Polyunsaturated Fatty Acid Enriched Diet Commands Fatty Acid Composition in Postnatal Brain and Protects from Neonatal Arterial Focal Stroke
Source: Transl Stroke Res. Author manuscript; Available in PMC 2023 Feb 1. (PMC9046339; doi:10.1007/s12975-021-00947-9)
Supplement: Chumak et al Suppl Material [file NIHMS1799223-supplement-Chumak_et_al_Suppl_Material.pdf]

Supplemental Fig. 1 Breeding characteristics in animals used for lipid and cytokine measurements

Maternal weight change over the course of pregnancy in animals fed standard (Stand., grey line), n−3 (blue line) or n−6 (yellow line) enriched diets (**a**). Number of pups per litter (**b**). Weight of pups at P10 (**c**).

Supplemental Fig. 2 Levels of fatty acid and chemokines differently affected by diet in male and female groups.

Levels of fatty acid 20:3 n-9 in blood plasma of male and female pups from mothers fed a standard (Stand.), n−3 enriched or a n−6 enriched diet (**a**). Levels of Eotaxin 2 and CCL22 in brain of males and females born to mothers fed a standard (Stand.), n−3 enriched or a n−6 enriched diet (**b**). Data presented as mean ± SD. One-way ANOVA with post-hoc Tukey's multiple comparison test was performed to compare groups within each sex. Individual p-values are listed within figures for data that are significantly different.

Supplemental Fig. 3 MCAO outcome in male and female pups.

Volume of brain injury 72 hours after tMCAO reperfusion in male and female pups born to mothers fed a standard (grey), n−3 (blue) or n−6 (yellow) enriched diet (**a, b**). Spectrin cleavage 24 hours after reperfusion via caspase-3 -dependent (125KDa band) and calpain-dependent (150KDa band), in injured (ipsi) and non-injured (contra) brain cortex of male and female pups (**c**). Data presented as mean ± SD. Kruskal-Wallis test followed by Dunn's post-hoc test (**a**) or two-way ANOVA followed by Tukey's post-hoc test (**c**) was performed to compare groups. Individual p-values are listed within figures for data that are significantly different.

Supplemental Table 1. Levels of phospholipid fatty acids in blood plasma of the offspring of mothers fed different diets along with p-values of statistical tests between diets (one-way ANOVA, Welch's ANOVA or Kruskal-Wallis as appropriate).

Supplemental Table 2. Total fatty acid lipids in brain of the offspring of mothers fed different diets along with p-values of statistical tests between diets (one-way ANOVA, Welch's ANOVA or Kruskas-Wallis as appropriate).

Supplemental Table 3. Levels of cytokines and chemokines in blood plasma of the offspring of mothers fed different diets along with p-values of statistical tests between diets (one-way ANOVA or Kruskas-Wallis as appropriate).

Supplemental Table 4. Levels of cytokines and chemokines in the brain of the offspring of mothers fed different diets along with p-values of statistical tests between diets (one-way ANOVA or Kruskas-Wallis as appropriate).

Supplemental Table 1. Levels of phospholipid fatty acids in blood plasma of the offspring of mothers fed different diets along with p-values of statistical tests between diets (one-way ANOVA, Welch's ANOVA or Kruskal-Wallis as appropriate).

**Fatty Acid**	**Mean±SD (Weight %)**			**p-values**		
	**Stand.**	**n-3**	**n-6**	**Stand./n−3**	**Stand./n−6**	**n−3/n−6**
**18:3 n−3 (ALA)**	0.07±0.01	0.06±0.02	0.02±0.01	0.085	**<0.001**	**<0.001**
**20:5 n−3 (EPA)**	0.20±0.05	5.66±1.90	0.03±0.01	**<0.001**	**<0.001**	**<0.001**
**22:5 n−3 (DPA)**	0.24±0.02	0.72±0.13	0.10±0.03	**<0.001**	**<0.001**	**<0.001**
**22:6 n−3 (DHA)**	6.77±0.98	11.48±0.93	2.51±0.56	**<0.001**	**<0.001**	**<0.001**
**18:2 n−6 (LA)**	26.50±2.15	17.00±3.40	23.89±3.43	**<0.001**	0.119	**<0.001**
**18:3 n−6**	0.15±0.01	0.11±0.01	0.21±0.01	**0.006**	0.1	**<0.001**
**20:2 n−6**	1.56±0.44	0.57±0.23	1.31±0.26	**<0.001**	0.178	**<0.001**
**20:3 n−6**	2.84±0.49	2.24±0.60	2.60±0.55	**0.033**	0.557	0.244
**20:4 n−6 (AA)**	11.07±2.15	5.85±1.03	13.63±1.13	**<0.001**	**0.006**	**<0.001**
**22:4 n−6**	0.10±0.03	0.04±0.01	0.29±0.09	**0.033**	**<0.001**	**<0.001**
**22:5 n−6**	0.12±0.03	0.06±0.01	1.68±0.16	**<0.001**	**<0.001**	**<0.001**
**14:0**	0.49±0.33	0.53±0.28	0.60±0.21	0.939	0.615	0.817
**16:0**	25.90±5.00	29.33±2.85	28.38±2.00	0.149	0.337	0.730
**17:0**	0.15±0.02	0.27±0.03	0.15±0.01	**<0.001**	1	**<0.001**
**18:0**	15.70±1.40	14.83±0.75	14.85±1.04	0.171	0.186	1
**20:0**	0.11±0.02	0.10±0.01	0.10±0.01	0.080	0.399	0.652
**22:0**	0.11±0.03	0.10±0.02	0.10±0.02	0.876	0.454	0.746
**23:0**	0.05±0.02	0.05±0.01	0.04±0.01	0.992	0.162	**0.013**
**24:0**	0.32±0.13	0.30±0.08	0.25±0.08	0.865	0.265	0.537
**16:1 n−7**	0.23±0.05	0.61±0.11	0.26±0.03	1.000	1.000	**<0.001**
**18:1 n−7**	1.15±0.19	1.59±0.20	1.10±0.06	**<0.001**	0.690	**<0.001**
**18:1 n−9**	3.39±0.40	5.98±1.61	4.48±1.02	0.227	**0.012**	0.394
**16:1 n−9**	0.08±0.03	0.10±0.02	0.11±0.03	**0.000**	**0.011**	**0.040**
**20:1 n−9**	0.26±0.07	0.18±0.05	0.28±0.05	**0.004**	0.561	**<0.001**
**20:3 n−9**	0.05±0.02	0.05±0.01	0.06±0.01	0.985	0.050	**<0.001**
**22:1 n−9**	0.08±0.05	0.63±0.02	0.07±0.03	1	1	1
**24:1 n−9**	1.32±0.72	1.04±0.32	1.13±0.38	0.381	0.630	0.918

Supplemental Table 2. Total lipid fatty acids in brain of the offspring of mothers fed different diets along with p-values of statistical tests between diets (one-way ANOVA, Welch's ANOVA or Kruskal-Wallis as appropriate).

Fatty Acid	Mean±SD (Weight %)			p-values		
	Stand.	n-3	n-6	Stand./n−3	Stand./n−6	n−3/n−6
**18:3 n−3 (ALA)**	0.02±0	0.017±0	0.016±0	0.081	**0.032**	0.909
**20:5 n−3 (EPA)**	0.04±0.01	0.51±0.09	0.01±0	**<0.001**	**<0.001**	**<0.001**
**22:5 n−3 (DPA)**	0.20±0.03	0.76±0.04	0.09±0.01	**0.016**	**0.016**	**<0.001**
**22:6 n−3 (DHA)**	15.49±1.67	19.46±1.00	8.85±0.69	**<0.001**	**<0.001**	**<0.001**
**18:2 n−6 (LA)**	0.83±0.04	0.74±0.06	0.76±0.08	**0.005**	**0.019**	0.867
**18:3 n−6**	0.06±0.01	0.04±0	0.07±0.01	**<0.001**	**0.023**	**<0.001**
**20:2 n−6**	0.21±0.01	0.13±0.01	0.20±0.02	**<0.001**	0.146	**<0.001**
**20:3 n−6**	0.63±0.06	0.98±0.05	0.48±0.04	**<0.001**	**<0.001**	**<0.001**
**20:4 n−6 (AA)**	14.91±1.13	9.59±0.57	16.29±1.61	**<0.001**	**0.019**	**<0.001**
**22:4 n−6**	1.64±0.36	0.52±0.20	2.00±0.33	**0.002**	0.36	**<0.001**
**22:5 n−6**	0.62±0.06	0.14±0.03	3.97±0.23	**<0.001**	**<0.001**	**<0.001**
**14:0**	1.36±0.41	1.62±0.40	1.59±0.52	0.327	0.416	0.984
**16:0**	28.46±1.04	29.50±1.39	29.60±1.90	0.211	0.160	0.987
**17:0**	2.22±2.63	2.19±2.57	2.09±2.49	1	0.543	0.243
**18:0**	17.17±1.01	16.63±0.88	17.13±1.13	0.399	0.994	0.460
**20:0**	0.08±0.01	0.12±0.10	0.08±0.01	0.930	0.882	0.994
**22:0**	0.07±0.01	0.07±0.01	0.06±0	0.967	0.601	0.456
**23:0**	0.01±0	0.01±0	0.01±0	0.542	0.646	0.985
**24:0**	0.06±0.01	0.07±0.01	0.05±0	**0.031**	0.131	**<0.001**
**16:1 n−7**	1.42±0.11	1.64±0.18	1.57±0.13	**0.002**	**0.046**	0.421
**18:1 n−7**	2.04±0.08	2.05±0.09	2.18±0.09	0.998	**0.002**	**0.002**
**16:1 n−9**	1.45±0.12	1.37±0.11	1.67±0.17	0.395	**<0.001**	**<0.001**
**18:1 n−9**	10.17±0.39	11.03±0.41	10.36±0.49	**<0.001**	0.539	**0.002**
**20:1 n−9**	0.21±0.01	0.25±0.01	0.21±0.01	**<0.001**	0.920	**<0.001**
**20:3 n−9**	0.16±0.02	0.24±0.03	0.17±0.03	**<0.001**	0.314	**<0.001**
**22:1 n−9**	0.28±0.34	0.22±0.26	0.28±0.33	1	1	1
**24:1 n−9**	0.09±0.01	0.89±0.01	0.09±0.01	0.949	0.992	0.981

Supplemental Table 3. Levels of cytokines and chemokines in blood plasma of the offspring of mothers fed different diets along with p-values of statistical tests between diets (one-way ANOVA).

Cytokine/ chemokine	Mean±SD (pg/ml)			p-values		
	Stand.	n-3	n-6	Stand./ n–3	Stand./ n–6	n–3/ n–6
**IL1b**	296.90±110.40	302.10±114.50	276.20±56.87	0.991	0.863	0.794
**IL2**	11.79±6.34	12.81±6.67	11.35±4.05	0.914	0.980	0.832
**IL4**	51.40±11.10	49.57±19.04	53.83±13.66	0.954	0.921	0.769
**IL6**	68.28±29.47	72.43±44.81	65.09±15.3	0.956	0.977	0.875
**IL10**	650.80±292.50	768.10±497.50	553.50±247.10	0.897	0.836	0.548
**IL16**	636.70±152.10	722.40±200.60	647.80±104.20	0.437	0.986	0.516
**INFg**	119.20±50.75	136.00±78.81	124.90±49.92	0.803	0.975	0.901
**GM-CSF**	3.65±2.32	3.76±2.02	4.01±1.57	0.989	0.894	0.949
**TNFa**	111.50±50.61	112.80±64.12	98.54±35.38	0.112	0.996	0.122
**CCL1**	59.75±14.50	48.65±9.83	59.28±13.87	0.952	0.463	0.640
**CCL2/MCP-1**	368.20±150.20	351.90±157.20	301.00±76.23	0.952	0.463	0.640
**CCL3/MIP1a**	10.77±5.01	12.09±7.74	9.18±3.20	0.854	0.803	0.454
**CCL4/MIP1b**	113.50±41.01	117.40±50.38	109.70±33.74	0.973	0.975	0.897
**CCL5/RANTES**	45.59±13.64	55.08±20.64	52.82±11.55	0.362	0.562	0.939
**CCL7/MCP-3**	130.30±46.16	135.30±20.77	134.70±39.27	0.951	0.959	0.999
**CCL11/Eotaxin**	329.60±152.80	285.20±110.00	297.80±78.01	0.663	0.800	0.968
**CCL12**	28.53±7.29	33.55±7.32	32.08±7.96	0.277	0.518	0.892
**CCL17**	108.40±32.06	137.10±81.33	120.80±43.94	1	1	1
**CCL19/MIP3b**	1360.00±343.30	1376.00±545.50	1321.00±201.40	0.995	0.976	0.950
**CCL20/MIP3a**	78.94±30.36	65.04±16.24	86.26±26.30	0.372	0.755	0.110
**CCL22**	200.80±55.11	170.60±37.46	230.90±73.90	0.503	0.451	0.071
**CCL24/Eotaxin2**	3891.00±1065.00	3777.00±1875.00	3763.00±985.20	0.977	0.972	1.000
**CCL27**	3487.00±1762.00	3397.00±1547.00	5223.00±2529.00	0.994	0.115	0.085
**CXCL1/KC**	143.20±58.45	153.70±88.44	126.10±32.95	0.925	0.821	0.574
**CXCL5**	2455.00±2064.00	4121.00±3853.00	699.20±191.80	1	0.156	**0.019**
**CXCL10**	768.50±201.20	808.00±245.30	781.10±127.20	0.883	0.988	0.944
**CXCL11**	580.30±151.10	594.60±271.00	593.50±173.00	0.986	0.989	1
**CXCL12**	722.80±153.80	898.70±330.20	797.90±240.70	0.268	0.771	0.614
**CXCL16**	534.20±222.60	533.90±150.30	668.00±211.00	1	0.235	0.234
**CXCL13/BCA1**	674.40±244.70	759.80±350.60	739.80±219.90	0.759	0.843	0.985
**CX3CL1/ Fraktalkine**	334.80±48.07	329.60±56.44	344.30±33.26	0.963	0.898	0.767

Supplemental Table 4. Levels of cytokines and chemokines in the brain of the offspring of mothers fed different diets along with p-values of statistical tests between diets (one-way ANOVA).

Cytokine/ chemokine	Mean±SD (pg/mg)			p-values		
	Stand.	n-3	n-6	Stand./n–3	Stand./n–6	n–3/n–6
**IL1b**	52.60±10.74	39.50±5.43	41.20±9.10	**0.003**	**0.009**	0.886
**IL2**	6.32±2.33	4.46±0.95	4.15±1.28	**0.022**	**0.007**	0.892
**IL4**	11.22±2.23	8.15±1.18	8.89±2.11	**0.001**	**0.013**	0.610
**IL6**	5.66±1.40	3.96±0.65	4.05±1.10	**0.002**	**0.003**	0.974
**IL10**	39.15±20.22	19.73±8.39	27.83±15.17	**0.022**	0.346	0.317
**IL16**	312.10±62.31	269.00±64.35	267.70±56.54	0.210	0.193	0.999
**INFg**	16.90±3.65	11.89±1.87	13.56±3.30	**0.001**	**0.032**	0.375
**GM-CSF**	0.26±0.08	0.20±0.05	0.20±0.05	0.054	0.074	0.988
**TNFa**	31.28±6.23	24.30±3.01	26.02±5.37	**0.006**	**0.044**	0.682
**CCL1**	5.01±1.24	3.55±0.54	3.86±0.89	**0.002**	**0.013**	0.699
**CCL2/MCP-1**	30.10±5.12	17.96±6.00	21.01±7.36	**<0.001**	**0.01**	0.538
**CCL3/MIP1a**	5.86±1.15	5.35±0.81	5.99±1.08	0.466	0.944	0.281
**CCL4/MIP1b**	10.42±2.47	7.48±1.69	7.75±2.56	**0.014**	**0.023**	0.958
**CCL5/RANTES**	3.99±0.90	2.70±1.13	2.64±0.73	**0.008**	**0.004**	0.981
**CCL7/MCP-3**	8.10±1.41	6.68±1.17	7.19±0.90	**0.020**	0.169	0.546
**CCL11/Eotaxin**	9.29± 2.04	7.95±1.68	7.45±1.29	0.154	**0.035**	0.747
**CCL12**	3.76±1.26	3.01±0.42	3.83±1.05	0.195	0.981	0.129
**CCL17**	15.66±3.21	12.67±2.30	14.26±2.22	**0.025**	0.410	0.301
**CCL19/MIP3b**	300.10±57.07	245.20±28.61	240.80±38.61	**0.011**	**0.006**	0.965
**CCL20/MIP3a**	7.06±1.70	5.26±1.01	5.22±0.88	**0.004**	**0.003**	0.997
**CCL22**	2.71±0.53	2.02±0.32	2.21±0.44	**0.008**	0.087	1
**CCL24/Eotaxin2**	1130.00±245.50	825.60±158.90	765.80±86.28	**0.041**	**0.003**	1
**CCL27**	558.80±128.30	420.70±65.93	455.00±80.05	**0.003**	**0.031**	0.656
**CXCL1/KC**	6.69±1.76	4.59±1.09	5.14±1.28	**0.002**	**0.028**	0.608
**CXCL5**	64.25±14.68	51.83±10.96	52±11.33	0.051	0.055	0.999
**CXCL10**	213.70±48.62	167.50±25.77	177.60±28.51	**0.009**	**0.048**	0.770
**CXCL11**	202.40±33.30	167.40±48.46	170.60±40.77	0.111	0.159	0.980
**CXCL12**	181.60±39.85	140.60±21.53	151.50±27.22	**0.007**	0.054	0.658
**CXCL16**	8.73±1.48	7.08±0.88	7.23±0.85	**0.003**	**0.007**	0.941
**CXCL13/BCA1**	158.20±32.80	119.90±14.33	123.90±17.14	**0.001**	**0.003**	0.901
**CX3CL1/ Fraktalkine**	381.40±68.79	315.00±47.50	329.40±39.64	**0.012**	0.058	0.789

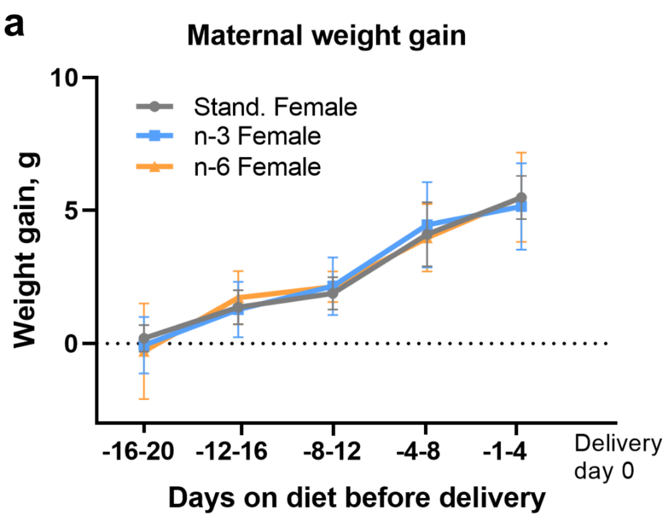
a
Maternal weight gain
Stand. Female
n-3 Female
n-6 Female
Weight gain, g
10
5
0
-16-20
-12-16
-8-12
-4-8
-1-4
Delivery day 0
Days on diet before delivery
b
Number of pups per litter
No. of pups per litter
10
8
6
4
2
0
Stand.
n-3
n-6
c
Weight of pups
Weight, g
8
6
4
2
0
p<0.001
p=0.005
Stand.
n-3
n-6

Suppl. Fig 2

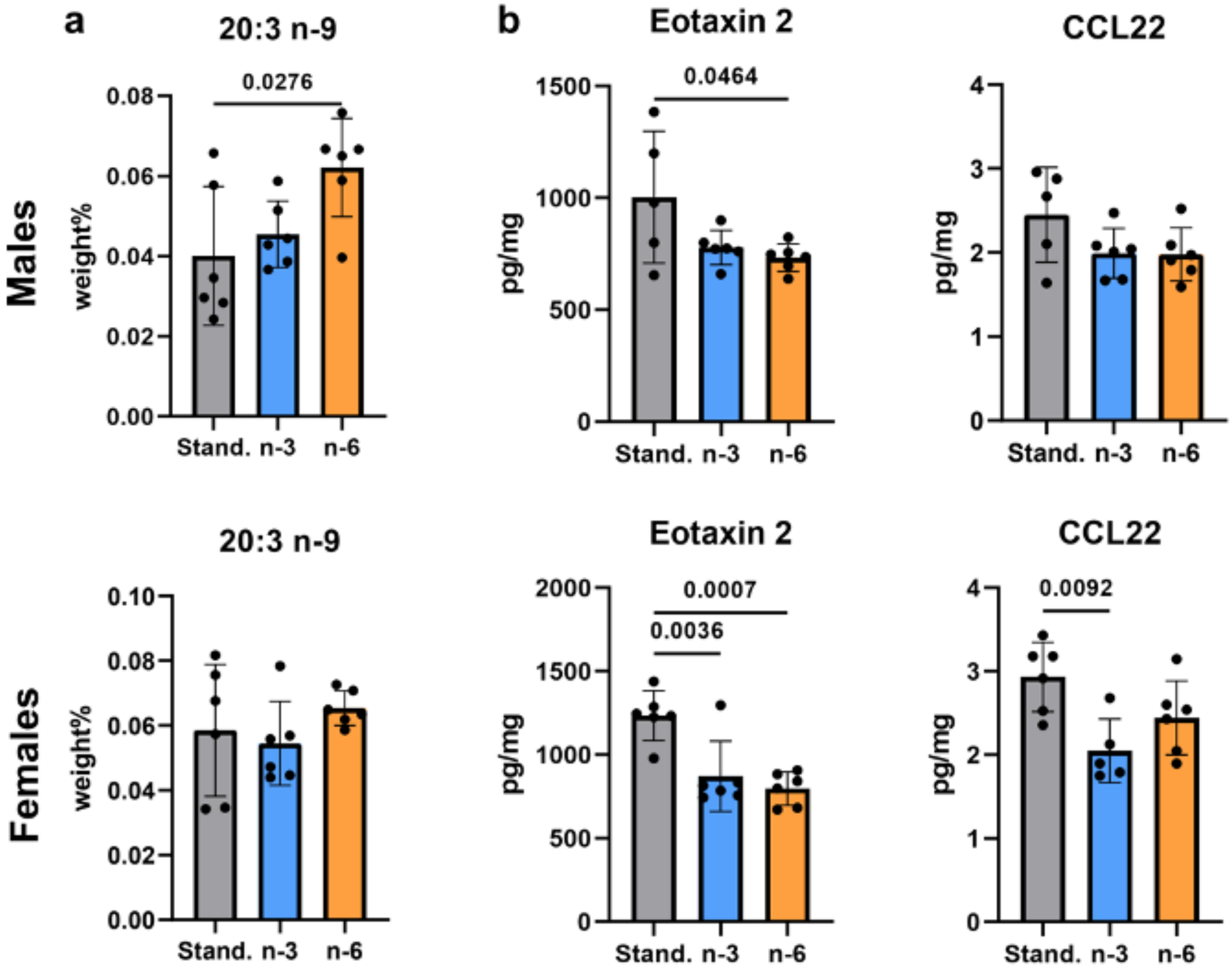
a
b
Males
Females
20:3 n-9
weight%
0.0276
Eotaxin 2
pg/mg
0.0464
CCL22
0.0007
0.0036
0.0092
Stand.
n-3
n-6

Suppl. Fig 3

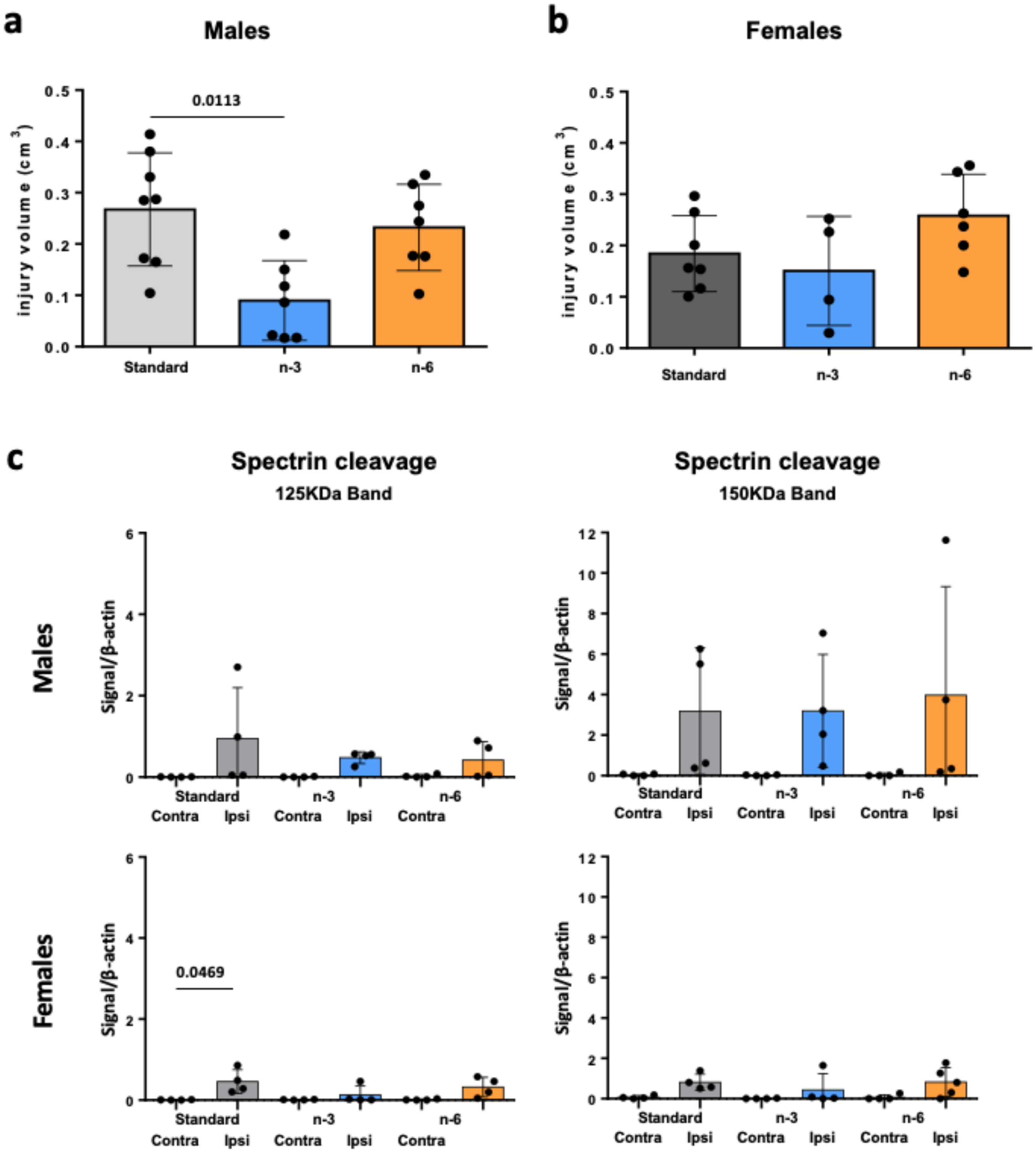
a
Males
0.0113
injury volume (cm^3)
0.0
0.1
0.2
0.3
0.4
0.5
Standard
n-3
n-6
b
Females
injury volume (cm^3)
0.0
0.1
0.2
0.3
0.4
0.5
Standard
n-3
n-6
c
Spectrin cleavage
125KDa Band
Spectrin cleavage
150KDa Band
Males
Females
Signal/β-actin
0
2
4
6
8
10
12
0.0469
Standard
n-3
n-6
Contra
Ipsi
