# Supplementary material for: Maternal n-3 Polyunsaturated Fatty Acid Enriched Diet Commands Fatty Acid Composition in Postnatal Brain and Protects from Neonatal Arterial Focal Stroke
Source: Transl Stroke Res. 2021 Oct 21;13(3):449–61. doi: 10.1007/s12975-021-00947-9 (PMC9046339; doi:10.1007/s12975-021-00947-9)
Supplement: Supplementary file 1 [file 12975_2021_947_MOESM1_ESM.docx]

**Supplemental Fig. 1 Breeding characteristics in animals used for lipid and cytokine measurements**

Maternal weight change over the course of pregnancy in animals fed standard (Stand., grey line), n−3 (blue line) or n−6 (yellow line) enriched diets (**a**). Number of pups per litter (**b**). Weight of pups at P10 (**c**). 

**Supplemental Fig. 2 Levels of fatty acid and chemokines differently affected by diet in male and female groups.** 

Levels of fatty acid 20:3 n-9 in blood plasma of male and female pups from mothers fed a standard (Stand.), n−3 enriched or a n−6 enriched diet (**a**). Levels of Eotaxin 2 and CCL22 in brain of males and females born to mothers fed a standard (Stand.), n−3 enriched or a n−6 enriched diet (**b**). Data presented as mean ± SD. One-way ANOVA with post-hoc Tukey’s multiple comparison test was performed to compare groups within each sex. Individual p-values are listed within figures for data that are significantly different.

**Supplemental Fig. 3 MCAO outcome in male and female pups.** 

Volume of brain injury 72 hours after tMCAO reperfusion in male and female pups born to mothers fed a standard (grey), n−3 (blue) or n−6 (yellow) enriched diet (**a, b**). Spectrin cleavage 24 hours after reperfusion via caspase-3 -dependent (125KDa band) and calpain-dependent (150KDa band), in injured (ipsi) and non-injured (contra) brain cortex of male and female pups (**c**). Data presented as mean ± SD. Kruskal-Wallis test followed by Dunn’s post-hoc test (**a**) or two-way ANOVA followed by Tukey’s post-hoc test (**c**) was performed to compare groups. Individual p-values are listed within figures for data that are significantly different.

**Supplemental Table 1**. Levels of phospholipid fatty acids in blood plasma of the offspring of mothers fed different diets along with p-values of statistical tests between diets (one-way ANOVA, Welch’s ANOVA or Kruskal-Wallis as appropriate).

**Supplemental Table 2.** Total fatty acid lipids in brain of the offspring of mothers fed different diets along with p-values of statistical tests between diets (one-way ANOVA, Welch’s ANOVA or Kruskas-Wallis as appropriate).

**Supplemental Table 3**. Levels of cytokines and chemokines in blood plasma of the offspring of mothers fed different diets along with p-values of statistical tests between diets (one-way ANOVA or Kruskas-Wallis as appropriate).

**Supplemental Table 4**. Levels of cytokines and chemokines in the brain of the offspring of mothers fed different diets along with p-values of statistical tests between diets (one-way ANOVA or Kruskas-Wallis as appropriate).
