# Supplementary material for: Maternal n-3 Polyunsaturated Fatty Acid Enriched Diet Commands Fatty Acid Composition in Postnatal Brain and Protects from Neonatal Arterial Focal Stroke
Source: Transl Stroke Res. 2021 Oct 21;13(3):449–61. doi: 10.1007/s12975-021-00947-9 (PMC9046339; doi:10.1007/s12975-021-00947-9)
Supplement: Supplementary file 2 [file 12975_2021_947_MOESM2_ESM.docx]

| **Fatty Acid** | **Mean±SD (Weight %)** |  |  | **p-values** |  |  |
| --- | --- | --- | --- | --- | --- | --- |
|  | **Stand.** | **n-3** | **n-6** | **Stand./n−3** | **Stand./n−6** | **n−3/n−6** |
| **18:3 n−3 (ALA)** | 0.07±0.01 | 0.06±0.02 | 0.02±0.01 | 0.085 | **<0.001** | **<0.001** |
| **20:5 n−3 (EPA)** | 0.20±0.05 | 5.66±1.90 | 0.03±0.01 | **<0.001** | **<0.001** | **<0.001** |
| **22:5 n−3 (DPA)** | 0.24±0.02 | 0.72±0.13 | 0.10±0.03 | **<0.001** | **<0.001** | **<0.001** |
| **22:6 n−3 (DHA)** | 6.77±0.98 | 11.48±0.93 | 2.51±0.56 | **<0.001** | **<0.001** | **<0.001** |
| **18:2 n−6 (LA)** | 26.50±2.15 | 17.00±3.40 | 23.89±3.43 | **<0.001** | 0.119 | **<0.001** |
| **18:3 n−6** | 0.15±0.01 | 0.11±0.01 | 0.21±0.01 | **0.006** | 0.1 | **<0.001** |
| **20:2 n−6** | 1.56±0.44 | 0.57±0.23 | 1.31±0.26 | **<0.001** | 0.178 | **<0.001** |
| **20:3 n−6** | 2.84±0.49 | 2.24±0.60 | 2.60±0.55 | **0.033** | 0.557 | 0.244 |
| **20:4 n−6 (AA)** | 11.07±2.15 | 5.85±1.03 | 13.63±1.13 | **<0.001** | **0.006** | **<0.001** |
| **22:4 n−6** | 0.10±0.03 | 0.04±0.01 | 0.29±0.09 | **0.033** | **<0.001** | **<0.001** |
| **22:5 n−6** | 0.12±0.03 | 0.06±0.01 | 1.68±0.16 | **<0.001** | **<0.001** | **<0.001** |
| **14:0** | 0.49±0.33 | 0.53±0.28 | 0.60±0.21 | 0.939 | 0.615 | 0.817 |
| **16:0** | 25.90±5.00 | 29.33±2.85 | 28.38±2.00 | 0.149 | 0.337 | 0.730 |
| **17:0** | 0.15±0.02 | 0.27±0.03 | 0.15±0.01 | **<0.001** | 1 | **<0.001** |
| **18:0** | 15.70±1.40 | 14.83±0.75 | 14.85±1.04 | 0.171 | 0.186 | 1 |
| **20:0** | 0.11±0.02 | 0.10±0.01 | 0.10±0.01 | 0.080 | 0.399 | 0.652 |
| **22:0** | 0.11±0.03 | 0.10±0.02 | 0.10±0.02 | 0.876 | 0.454 | 0.746 |
| **23:0** | 0.05±0.02 | 0.05±0.01 | 0.04±0.01 | 0.992 | 0.162 | **0.013** |
| **24:0** | 0.32±0.13 | 0.30±0.08 | 0.25±0.08 | 0.865 | 0.265 | 0.537 |
| **16:1 n−7** | 0.23±0.05 | 0.61±0.11 | 0.26±0.03 | 1.000 | 1.000 | **<0.001** |
| **18:1 n−7** | 1.15±0.19 | 1.59±0.20 | 1.10±0.06 | **<0.001** | 0.690 | **<0.001** |
| **18:1 n−9** | 3.39±0.40 | 5.98±1.61 | 4.48±1.02 | 0.227 | **0.012** | 0.394 |
| **16:1 n−9** | 0.08±0.03 | 0.10±0.02 | 0.11±0.03 | **0.000** | **0.011** | **0.040** |
| **20:1 n−9** | 0.26±0.07 | 0.18±0.05 | 0.28±0.05 | **0.004** | 0.561 | **<0.001** |
| **20:3 n−9** | 0.05±0.02 | 0.05±0.01 | 0.06±0.01 | 0.985 | 0.050 | **<0.001** |
| **22:1 n−9** | 0.08±0.05 | 0.63±0.02 | 0.07±0.03 | 1 | 1 | 1 |
| **24:1 n−9** | 1.32±0.72 | 1.04±0.32 | 1.13±0.38 | 0.381 | 0.630 | 0.918 |

**Supplemental Table 1.** Levels of phospholipid fatty acids in blood plasma of the offspring of mothers fed different diets along with p-values of statistical tests between diets (one-way ANOVA, Welch’s ANOVA or Kruskal-Wallis as appropriate). 

**Supplemental Table 2.** Total lipid fatty acids in brain of the offspring of mothers fed different diets along with p-values of statistical tests between diets (one-way ANOVA, Welch’s ANOVA or Kruskal-Wallis as appropriate).

| **Fatty Acid** | **Mean±SD (Weight %)** |  |  | **p-values** |  |  |
| --- | --- | --- | --- | --- | --- | --- |
|  | **Stand.** | **n-3** | **n-6** | **Stand./n−3** | **Stand./n−6** | **n−3/n−6** |
| **18:3 n−3 (ALA)** | 0.02±0 | 0.017±0 | 0.016±0 | 0.081 | **0.032** | 0.909 |
| **20:5 n−3 (EPA)** | 0.04±0.01 | 0.51±0.09 | 0.01±0 | **<0.001** | **<0.001** | **<0.001** |
| **22:5 n−3 (DPA)** | 0.20±0.03 | 0.76±0.04 | 0.09±0.01 | **0.016** | **0.016** | **<0.001** |
| **22:6 n−3 (DHA)** | 15.49±1.67 | 19.46±1.00 | 8.85±0.69 | **<0.001** | **<0.001** | **<0.001** |
| **18:2 n−6 (LA)** | 0.83±0.04 | 0.74±0.06 | 0.76±0.08 | **0.005** | **0.019** | 0.867 |
| **18:3 n−6** | 0.06±0.01 | 0.04±0 | 0.07±0.01 | **<0.001** | **0.023** | **<0.001** |
| **20:2 n−6** | 0.21±0.01 | 0.13±0.01 | 0.20±0.02 | **<0.001** | 0.146 | **<0.001** |
| **20:3 n−6** | 0.63±0.06 | 0.98±0.05 | 0.48±0.04 | **<0.001** | **<0.001** | **<0.001** |
| **20:4 n−6 (AA)** | 14.91±1.13 | 9.59±0.57 | 16.29±1.61 | **<0.001** | **0.019** | **<0.001** |
| **22:4 n−6** | 1.64±0.36 | 0.52±0.20 | 2.00±0.33 | **0.002** | 0.36 | **<0.001** |
| **22:5 n−6** | 0.62±0.06 | 0.14±0.03 | 3.97±0.23 | **<0.001** | **<0.001** | **<0.001** |
| **14:0** | 1.36±0.41 | 1.62±0.40 | 1.59±0.52 | 0.327 | 0.416 | 0.984 |
| **16:0** | 28.46±1.04 | 29.50±1.39 | 29.60±1.90 | 0.211 | 0.160 | 0.987 |
| **17:0** | 2.22±2.63 | 2.19±2.57 | 2.09±2.49 | 1 | 0.543 | 0.243 |
| **18:0** | 17.17±1.01 | 16.63±0.88 | 17.13±1.13 | 0.399 | 0.994 | 0.460 |
| **20:0** | 0.08±0.01 | 0.12±0.10 | 0.08±0.01 | 0.930 | 0.882 | 0.994 |
| **22:0** | 0.07±0.01 | 0.07±0.01 | 0.06±0 | 0.967 | 0.601 | 0.456 |
| **23:0** | 0.01±0 | 0.01±0 | 0.01±0 | 0.542 | 0.646 | 0.985 |
| **24:0** | 0.06±0.01 | 0.07±0.01 | 0.05±0 | **0.031** | 0.131 | **<0.001** |
| **16:1 n−7** | 1.42±0.11 | 1.64±0.18 | 1.57±0.13 | **0.002** | **0.046** | 0.421 |
| **18:1 n−7** | 2.04±0.08 | 2.05±0.09 | 2.18±0.09 | 0.998 | **0.002** | **0.002** |
| **16:1 n−9** | 1.45±0.12 | 1.37±0.11 | 1.67±0.17 | 0.395 | **<0.001** | **<0.001** |
| **18:1 n−9** | 10.17±0.39 | 11.03±0.41 | 10.36±0.49 | **<0.001** | 0.539 | **0.002** |
| **20:1 n−9** | 0.21±0.01 | 0.25±0.01 | 0.21±0.01 | **<0.001** | 0.920 | **<0.001** |
| **20:3 n−9** | 0.16±0.02 | 0.24±0.03 | 0.17±0.03 | **<0.001** | 0.314 | **<0.001** |
| **22:1 n−9** | 0.28±0.34 | 0.22±0.26 | 0.28±0.33 | 1 | 1 | 1 |
| **24:1 n−9** | 0.09±0.01 | 0.89±0.01 | 0.09±0.01 | 0.949 | 0.992 | 0.981 |

**Supplemental Table 3.** Levels of cytokines and chemokines in blood plasma of the offspring of mothers fed different diets along with p-values of statistical tests between diets (one-way ANOVA).

| **Cytokine/** **chemokine** | **Mean±SD (pg/ml)** |  |  | **p-values** |  |  |
| --- | --- | --- | --- | --- | --- | --- |
|  | **Stand.** | **n-3** | **n-6** | **Stand./n−3** | **Stand./** **n−6** | **n−3/** **n−6** |
| **IL1b** | 296.90±110.40 | 302.10±114.50 | 276.20±56.87 | 0.991 | 0.863 | 0.794 |
| **IL2** | 11.79±6.34 | 12.81±6.67 | 11.35±4.05 | 0.914 | 0.980 | 0.832 |
| **IL4** | 51.40±11.10 | 49.57±19.04 | 53.83±13.66 | 0.954 | 0.921 | 0.769 |
| **IL6** | 68.28±29.47 | 72.43±44.81 | 65.09±15.3 | 0.956 | 0.977 | 0.875 |
| **IL10** | 650.80±292.50 | 768.10±497.50 | 553.50±247.10 | 0.897 | 0.836 | 0.548 |
| **IL16** | 636.70±152.10 | 722.40±200.60 | 647.80±104.20 | 0.437 | 0.986 | 0.516 |
| **INFg** | 119.20±50.75 | 136.00±78.81 | 124.90±49.92 | 0.803 | 0.975 | 0.901 |
| **GM-CSF** | 3.65±2.32 | 3.76±2.02 | 4.01±1.57 | 0.989 | 0.894 | 0.949 |
| **TNFa** | 111.50±50.61 | 112.80±64.12 | 98.54±35.38 | 0.112 | 0.996 | 0.122 |
| **CCL1** | 59.75±14.50 | 48.65±9.83 | 59.28±13.87 | 0.952 | 0.463 | 0.640 |
| **CCL2/MCP-1** | 368.20±150.20 | 351.90±157.20 | 301.00±76.23 | 0.952 | 0.463 | 0.640 |
| **CCL3/MIP1a** | 10.77±5.01 | 12.09±7.74 | 9.18±3.20 | 0.854 | 0.803 | 0.454 |
| **CCL4/MIP1b** | 113.50±41.01 | 117.40±50.38 | 109.70±33.74 | 0.973 | 0.975 | 0.897 |
| **CCL5/RANTES** | 45.59±13.64 | 55.08±20.64 | 52.82±11.55 | 0.362 | 0.562 | 0.939 |
| **CCL7/MCP-3** | 130.30±46.16 | 135.30±20.77 | 134.70±39.27 | 0.951 | 0.959 | 0.999 |
| **CCL11/Eotaxin** | 329.60±152.80 | 285.20±110.00 | 297.80±78.01 | 0.663 | 0.800 | 0.968 |
| **CCL12** | 28.53±7.29 | 33.55±7.32 | 32.08±7.96 | 0.277 | 0.518 | 0.892 |
| **CCL17** | 108.40±32.06 | 137.10±81.33 | 120.80±43.94 | 1 | 1 | 1 |
| **CCL19/MIP3b** | 1360.00±343.30 | 1376.00±545.50 | 1321.00±201.40 | 0.995 | 0.976 | 0.950 |
| **CCL20/MIP3a** | 78.94±30.36 | 65.04±16.24 | 86.26±26.30 | 0.372 | 0.755 | 0.110 |
| **CCL22** | 200.80±55.11 | 170.60±37.46 | 230.90±73.90 | 0.503 | 0.451 | 0.071 |
| **CCL24/Eotaxin2** | 3891.00±1065.00 | 3777.00±1875.00 | 3763.00±985.20 | 0.977 | 0.972 | 1.000 |
| **CCL27** | 3487.00±1762.00 | 3397.00±1547.00 | 5223.00±2529.00 | 0.994 | 0.115 | 0.085 |
| **CXCL1/KC** | 143.20±58.45 | 153.70±88.44 | 126.10±32.95 | 0.925 | 0.821 | 0.574 |
| **CXCL5** | 2455.00±2064.00 | 4121.00±3853.00 | 699.20±191.80 | 1 | 0.156 | **0.019** |
| **CXCL10** | 768.50±201.20 | 808.00±245.30 | 781.10±127.20 | 0.883 | 0.988 | 0.944 |
| **CXCL11** | 580.30±151.10 | 594.60±271.00 | 593.50±173.00 | 0.986 | 0.989 | 1 |
| **CXCL12** | 722.80±153.80 | 898.70±330.20 | 797.90±240.70 | 0.268 | 0.771 | 0.614 |
| **CXCL16** | 534.20±222.60 | 533.90±150.30 | 668.00±211.00 | 1 | 0.235 | 0.234 |
| **CXCL13/BCA1** | 674.40±244.70 | 759.80±350.60 | 739.80±219.90 | 0.759 | 0.843 | 0.985 |
| **CX3CL1/** **Fraktalkine** | 334.80±48.07 | 329.60±56.44 | 344.30±33.26 | 0.963 | 0.898 | 0.767 |

**Supplemental Table 4.** Levels of cytokines and chemokines in the brain of the offspring of mothers fed different diets along with p-values of statistical tests between diets (one-way ANOVA).

| **Cytokine/** **chemokine** | **Mean±SD (pg/mg)** |  |  | **p-values** |  |  |
| --- | --- | --- | --- | --- | --- | --- |
|  | **Stand.** | **n-3** | **n-6** | **Stand./n−3** | **Stand./n−6** | **n−3/n−6** |
| **IL1b** | 52.60±10.74 | 39.50±5.43 | 41.20±9.10 | **0.003** | **0.009** | 0.886 |
| **IL2** | 6.32±2.33 | 4.46±0.95 | 4.15±1.28 | **0.022** | **0.007** | 0.892 |
| **IL4** | 11.22±2.23 | 8.15±1.18 | 8.89±2.11 | **0.001** | **0.013** | 0.610 |
| **IL6** | 5.66±1.40 | 3.96±0.65 | 4.05±1.10 | **0.002** | **0.003** | 0.974 |
| **IL10** | 39.15±20.22 | 19.73±8.39 | 27.83±15.17 | **0.022** | 0.346 | 0.317 |
| **IL16** | 312.10±62.31 | 269.00±64.35 | 267.70±56.54 | 0.210 | 0.193 | 0.999 |
| **INFg** | 16.90±3.65 | 11.89±1.87 | 13.56±3.30 | **0.001** | **0.032** | 0.375 |
| **GM-CSF** | 0.26±0.08 | 0.20±0.05 | 0.20±0.05 | 0.054 | 0.074 | 0.988 |
| **TNFa** | 31.28±6.23 | 24.30±3.01 | 26.02±5.37 | **0.006** | **0.044** | 0.682 |
| **CCL1** | 5.01±1.24 | 3.55±0.54 | 3.86±0.89 | **0.002** | **0.013** | 0.699 |
| **CCL2/MCP-1** | 30.10±5.12 | 17.96±6.00 | 21.01±7.36 | **<0.001** | **0.01** | 0.538 |
| **CCL3/MIP1a** | 5.86±1.15 | 5.35±0.81 | 5.99±1.08 | 0.466 | 0.944 | 0.281 |
| **CCL4/MIP1b** | 10.42±2.47 | 7.48±1.69 | 7.75±2.56 | **0.014** | **0.023** | 0.958 |
| **CCL5/RANTES** | 3.99±0.90 | 2.70±1.13 | 2.64±0.73 | **0.008** | **0.004** | 0.981 |
| **CCL7/MCP-3** | 8.10±1.41 | 6.68±1.17 | 7.19±0.90 | **0.020** | 0.169 | 0.546 |
| **CCL11/Eotaxin** | 9.29± 2.04 | 7.95±1.68 | 7.45±1.29 | 0.154 | **0.035** | 0.747 |
| **CCL12** | 3.76±1.26 | 3.01±0.42 | 3.83±1.05 | 0.195 | 0.981 | 0.129 |
| **CCL17** | 15.66±3.21 | 12.67±2.30 | 14.26±2.22 | **0.025** | 0.410 | 0.301 |
| **CCL19/MIP3b** | 300.10±57.07 | 245.20±28.61 | 240.80±38.61 | **0.011** | **0.006** | 0.965 |
| **CCL20/MIP3a** | 7.06±1.70 | 5.26±1.01 | 5.22±0.88 | **0.004** | **0.003** | 0.997 |
| **CCL22** | 2.71±0.53 | 2.02±0.32 | 2.21±0.44 | **0.008** | 0.087 | 1 |
| **CCL24/Eotaxin2** | 1130.00±245.50 | 825.60±158.90 | 765.80±86.28 | **0.041** | **0.003** | 1 |
| **CCL27** | 558.80±128.30 | 420.70±65.93 | 455.00±80.05 | **0.003** | **0.031** | 0.656 |
| **CXCL1/KC** | 6.69±1.76 | 4.59±1.09 | 5.14±1.28 | **0.002** | **0.028** | 0.608 |
| **CXCL5** | 64.25±14.68 | 51.83±10.96 | 52±11.33 | 0.051 | 0.055 | 0.999 |
| **CXCL10** | 213.70±48.62 | 167.50±25.77 | 177.60±28.51 | **0.009** | **0.048** | 0.770 |
| **CXCL11** | 202.40±33.30 | 167.40±48.46 | 170.60±40.77 | 0.111 | 0.159 | 0.980 |
| **CXCL12** | 181.60±39.85 | 140.60±21.53 | 151.50±27.22 | **0.007** | 0.054 | 0.658 |
| **CXCL16** | 8.73±1.48 | 7.08±0.88 | 7.23±0.85 | **0.003** | **0.007** | 0.941 |
| **CXCL13/BCA1** | 158.20±32.80 | 119.90±14.33 | 123.90±17.14 | **0.001** | **0.003** | 0.901 |
| **CX3CL1/** **Fraktalkine** | 381.40±68.79 | 315.00±47.50 | 329.40±39.64 | **0.012** | 0.058 | 0.789 |
